# Computational and theoretical chemistry of newly synthesized and characterized 2,2^’^-(5,5^’^-(1,4-phenylene)bis(1*H*-tetrazole-5,1-diyl))bis-*N*-acetamides

**DOI:** 10.1186/s13065-023-01011-3

**Published:** 2023-08-14

**Authors:** Syeda Abida Ejaz, Aftab Farid, Seema Zargar, Pervaiz Ali Channar, Mubashir Aziz, Tanveer A. Wani, Hafiz Muhammad Attaullah, Rabail Ujhan, Arfa Tehzeeb, Aamer Saeed, Hafiz Saqib Ali, Mauricio F. Erben

**Affiliations:** 1 Department of Pharmaceutical Chemistry, Faculty of Pharmacy, The Islamia University of Bahawalpur, Bahawalpur, 63100 Pakistan; 2 Department of Chemistry, Quaid-I-Azam University Islamabad, Islamabad, 45320 Pakistan; 3 Department of Pharmaceutical Chemistry, College of Pharmacy, King Saud University, P.O. Box 2457, 11451 Riyadh, Saudi Arabia; 4 Department of Basic Sciences and Humanities, Faculty of of Information Science and Humanities, Dawood University of Engineering and Technology Karachi, Karachi, 74800 Pakistan; 5 Dr. M. A. Kazi Institute of Chemistry, University of Sindh, Jamshoro, 76080 Pakistan; 6 Department of Pharmacy, Quaid-I-Azam University Islamabad, Islamabad, 45320 Pakistan; 7 Manchester Institute of Biotechnology, The University of Manchester, 131 Princess St., Manchester, M1 7DN UK; 8 School of Chemistry, The University of Manchester, Oxford Road, Manchester, M13 9PL UK; 9 Departamento de Química, Facultad de Ciencias Exactas, CEQUINOR (UNLP, CONICET-CCT La Plata), Universidad Nacional de La Plata, C.C. 962 (1900) La Plata, República Argentina; 10 Department of Biochemistry, College of Science, King Saud University, P.O. Box 22452, 11451, Riyadh, Saudi Arabia

**Keywords:** Synthesis, Bis-tetrazole acetamides, DFT, Energetic materials, Thermodynamic properties, Aryl coupling synthons

## Abstract

Energetic heterocycles, including pyridines, triazoles, and tetrazoles, exhibit greater density, heats of formation, and oxygen balance compared to their carbocyclic counterparts, making them a promising approach for synthesizing novel bis-tetrazole acetamides. Synthesized compounds A-F, some of which feature a chlorine atom attached to the phenyl ring, serve as valuable synthons for aryl coupling reactions. Analysis via ^1^H-NMR and ^13^C-NMR spectroscopy, as well as density functional considerations through B3LYP functional correlation with 6-311 +  + G(d) and 6-31G(d) basis set, revealed the observed LUMO/HOMO energies and charge transfer within the molecule. Additionally, the dipole moment, chemical hardness, softness, ionization potential, local reactivity potential via Fukui indices and thermodynamic properties (entropy, enthalpy, and Gibbs free energy) of the molecule were calculated through density functional theory studies. In addition, Molecular Docking studies were conducted to investigate the anti-cancer potential of synthesized heterocyclic compounds against caspase 3, NF-KAPPA-B and P53 protein. Molecular docking analysis demonstrated a potent interaction between 2,2’-(5,5’-(1,4-phenylene)bis(1*H*-tetrazole-5,1-diyl))bis-N-(2,4-dinitrophenyl) acetamides **(6d)** and TP53 and NF-KAPPA-B with binding energies of − 11.8 kJ/mol and − 10.9 kJ/mol for TP53 and NF-KAPPA-B, respectively. Similarly, 2,2’-(5,5’–(1,4-phenylene)bis(1*H*-tetrazole-5,1-diyl))bis-N-(2-chlorophenyl) acetamides **(6f)** exhibited a strong interaction with caspase-3 with binding energy of -10.0 kJ/mol, indicating their potential as therapeutic agents against these proteins. Furthermore, the findings of current study was further strengthen by 100 ns molecular dynamics (MD) simulations. Finally, theoretical studies of oxygen balance and nitrogen percentage suggest that these molecules can be utilized as energetic materials.

**Graphical Abstract:**

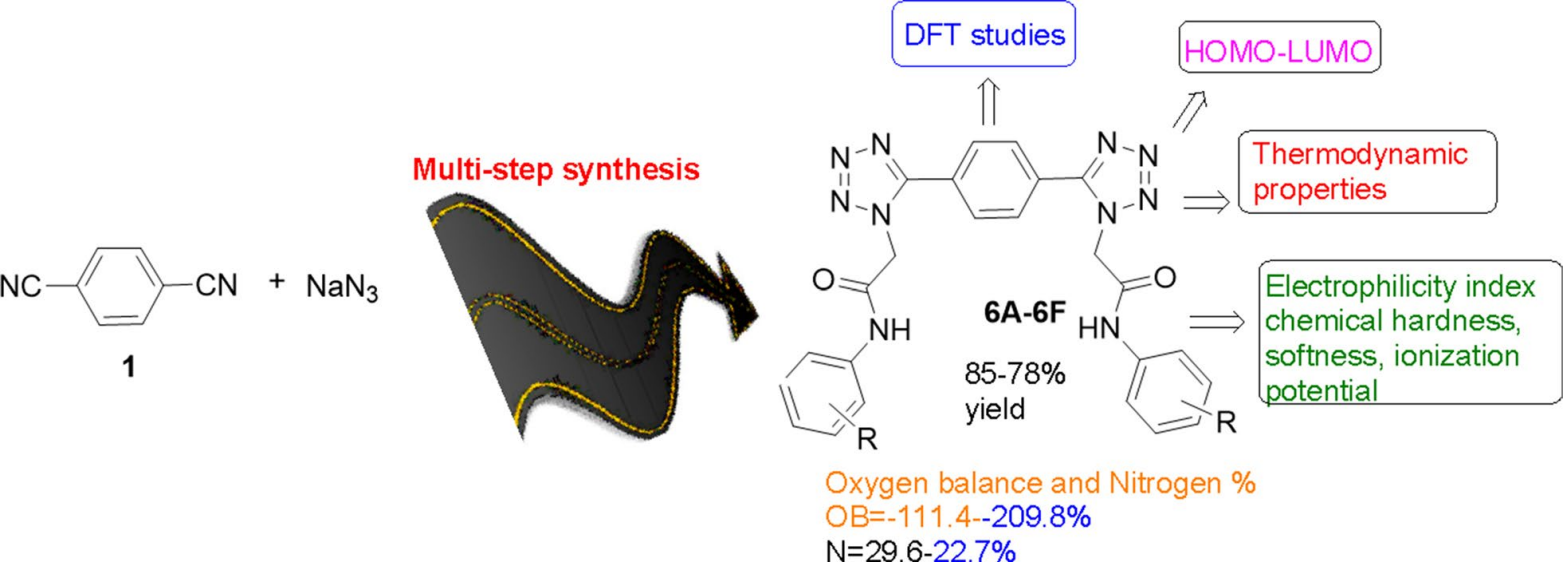

**Supplementary Information:**

The online version contains supplementary material available at 10.1186/s13065-023-01011-3.

## Introduction

Tetrazoles are four nitrogen and one carbon containing five membered important heterocyclic compounds. The field of heterocyclic chemistry has matured but the diverse applications of tetrazole heterocycles still seeks attention from the perspective of applied chemistry. Bladin in 1855 first time synthesized tetrazoles and since then the number of publications devoted to synthesis and applications of tetrazoles increase per annum [1]. In addition, tetrazoles possess greater heat of formation as they contain greater proportion of carbon–nitrogen and nitrogen-nitrogen bonds. The tetrazoles have low percentage of C and H bonds are highly insensitive towards electrostatic discharge, impulse or shock [2]. Tetrazoles nucleus is highly desired in energetic materials because it enhances density, keeps good oxygen balance ration and produced enormous gaseous molecules. Already described synthesis methods for 1,5-DSTs include the use of amides, nitriles, imidoyl chlorides, thioamides, amines, ketones, alkenes and hetero-cumulenes (e.g., isocyanates, carbodiimides, isothiocyanates, and ketenimines) as a precursor. Some other publications for the synthesis of 1,5-DSTs includes the transformation of the 1- and 5-substituted tetrazoles into 1,5-DSTs [3–11]. Significant development was accomplished by Sharpless and co-worker who described that in the presence of a zinc salt, nitriles efficiently react with sodium azide in water; however, for least-reactive nitriles, exceptional reaction conditions i.e. high Temperature (170 °C) for 48 h and sealed pressure reactor was required [3].

Tetrazole ring has interplayed key role in designing of various bioactive molecules against viral and microbial derived diseases [12, 13]. In pharmaceutical chemistry, tetrazoles used as surrogates of carboxylic acids and acts as spacers [14]. Tetrazoles have found applications in designing of energetic materials. Moreover, tetrazoles provide a perfect platform to carry out synthetic protocols to obtain desired heterocyclic units. Furthermore, Tetrazole containing five-membered heterocyclic compounds is a crucial pharmacophore fragment that has been utilized in the development of potential anti-cancer agents. Several compounds, including coordination compounds and natural compounds with tetrazole moiety, have been identified as prospective anticancer agents [15, 16]. These compounds have been found to inhibit the proliferation of cancer cells and induce apoptosis by targeting various pathways, including kappa caspase 3 and p53. Caspase 3, p53, and kappa protein are essential proteins involved in various cellular processes, including apoptosis, cell cycle regulation, and inflammation [17]. Dysregulation of these proteins has been implicated in the development of various diseases, including cancer. Caspase 3 is a protease that plays a crucial role in the apoptotic pathway. It is responsible for the cleavage of several proteins, leading to the programmed death of cells. Dysregulation of caspase 3 has been linked to cancer, Alzheimer's disease, and other disorders. P53 is a well-known tumor suppressor protein that regulates the cell cycle and prevents the development of cancer [18]. It plays a crucial role in inducing cell cycle arrest and apoptosis in response to DNA damage. Mutations in the p53 gene are one of the most common genetic alterations in human cancers. The nuclear factor kappa B (NF-κB) pathway is a critical signaling pathway involved in the regulation of inflammation, immunity, and cell survival. Dysregulation of NF-κB has been associated with several diseases, including cancer, inflammatory disorders, and autoimmune diseases [19]. So, targeting these tumor proteins via novel heterocyclic compounds can prove as promising approach in designing the novel anti-cancer agents.

In current study, we have synthesized the target compounds in a multistep synthesis route by following the well-known procedures and employing more reactive nitriles. In addition to the experimental synthesis and characterization of the synthesized compounds, we also explored their potential as anti-cancer agents through molecular docking studies. Specifically, we targeted critical proteins involved in cancer development, including caspase 3, p53, and NF-κB, to evaluate the binding affinities of the synthesized compounds [20]. Our molecular docking results indicated that some of the synthesized compounds showed strong binding affinity towards the targeted proteins, suggesting their potential as anti-cancer agents. These findings were complemented by our computational investigations, which provided important insights into the chemical reactivity, thermodynamic properties, and energetic properties of the synthesized molecules. Overall, the combination of experimental synthesis and computational analysis used in this study offers a promising approach to the development of novel anti-cancer agents. The computational investigations were carried out to undermine the salient features of the synthesized molecules and to give close insights about their chemical reactivity, thermodynamic properties and energetic properties.

## Methods and materials

### Substrates and reagents

2-Chloroaniline, 3-Chloroaniline, 4-Chloroaniline, 2,3-dichloroaniline, 2,4,6-trimethylaniline, 2,4-dinitroaniline, 1,4-dicyanobenzene, sodium azide, zinc chloride, ethyl chloroacetate, potassium carbonate, sodium hydroxide, thionyl chloride, potassium thiocyanate, mercuric chloride and triethyl amine were bought from Fluka, Alfa-aeser, Sigma-Aldrich and Merck and utilized without further purification. Solvents like DMF, DCM, acetone, ethanol, methanol, *n*-hexane, ethyl acetate were purchased from commercial stores.

### Purification of solvents and reagents

Standard protocols were used for the purification and drying of the solvents and reagents. A4 size molecular sieves were preheated at 500 ℃ for 8 h and all the dried solvents were preserved over these preheated molecular sieves.

## Chromatographic techniques

### Thin layer chromatography (TLC)

Aluminum plates coated with silica gel (layer thickness 0.2 mm, HF_254_, Reidal-Haen from Merck) was used to screen the reactions kinetics via thin layer chromatography, by using solvent systems*. Ultraviolet light (λmax 254 and 365 nm) was used to detect the chromatograms. Chloroform: Methanol (1:1), Chloroform: Methanol (8:2), *n*-Hexane: Ethyl acetate (1:1), *n*-Hexane: Ethyl acetate (4:1).

### Instruments Used

By using Stuart (SMP3) melting point apparatus, melting point was determined by open capillary method. Avance series 300 MHz purchased from Bruker was used to record the ^1^H-NMR in (CD_3_)_2_SO solvent and TMS as an internal reference.

Chemical shifts and *J* values are given in δ-scale (ppm) in Hz respectively. Abbreviations used for singlet, doublet, doublet of doublet, triplet and multiplet have been s, d, dd, t and m correspondingly. To get ^13^C-NMR spectra, 75.5 MHz NMR spectrometer in (CH_3_)_2_CO, CDCl_3_, and (CD_3_)_2_SO solvents is used.

### General procedure for compound 6a-f

**Scheme 1:**
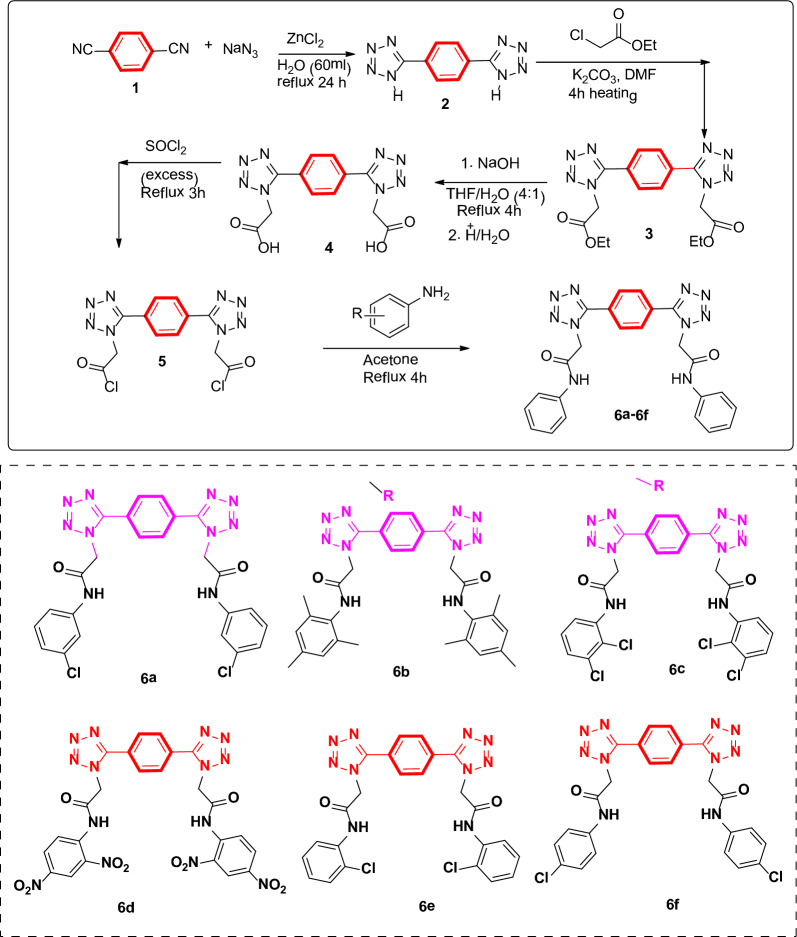
Synthesis of 2,2′-(5,5′- (1,4-phenylene)bis(1*H*-tetrazole-5,1-diyl))bis-*N*-(4-chlorophenyl) acetamide 6a-6f

The synthesis of bis-tetrazole acetamides is outlined in Scheme 1. Amides of bis-1,4-(1*H*-tetrazole) was afforded by using 1,4-dicyanobenzene as a starting material. In first step 5-(4-(1*H*-tetrazole-5-yl)phenyl)-1*H*-tetrazole **2** was synthesized by the reaction of 1,4-dicyanobenzene and NaN_3_ in water in the presence of ZnCl_2_ as a catalyst. Compound **3** was treated with ethyl chloro-acetate in potassium carbonate as a base to form diethyl 2,2′-(5,5′-(1,4-phenylene)bis(1*H*-tetrazole-5,1-diyl))diacetate **3.** compound **3** on hydrolysis give a respective acid which on further reaction with thionyl chloride followed by substituted anilines give 2,2′-(5,5′-(1,4-phenylene)bis(1*H*-tetrazole-5,1-diyl))bis-*N*-(substituted phenyl) acetamide **(6A-F)** all of the products were obtained in excellent yield.

#### 2,2^’^-(5,5^’^-(1,4-phenylene)bis(1*H*-tetrazole-5,1-diyl))bis-*N*-(3-chlorophenyl) acetamide: 6a

Pale yellow solid; Yield: 80%; Rf (chloroform: methanol, 8:2): 0.78; decomposition temperature (^o^C): 225; **FTIR (cm**^**−1**^**) 3186 (NH), 3035 (sp**^**2**^** CH), 2914 (sp**^**3**^** CH), 1677 (C = O),1587 (C = N),1477,(C = C)** ^1^H-NMR ((CD_3_))_2_SO, 300 MHz): δ 10.90 (s,2H, NH), 8.29 (s, 4H, Ar–H), 7.78 (s, 2H, Ar–H), 7.17–7.51 (m, 6H, Ar–H), 5.83 (s, 4H, CH_2_); ^13^C-NMR ((CD_3_))_2_SO, 75.5 MHz): δ 167.1, 163.8, 140.1, 133.7, 131.5, 129.2, 127.6, 124.3, 119.4, 118.3, 55.9. Anal. Calcd. for C_24_H_18_Cl_2_N_10_O_2_: C, 52.46; H, 3.31; N, 25.50; found: C, 52.44; H, 3.34; N, 25.53. HRMS Caled for C_24_H_18_Cl_2_N_10_O_2_ + H: 548.0991. Found 548.0989.

#### 2,2'-(5,5'-(1,4-phenylene)bis(1H-tetrazole-5,1-diyl))bis(N-mesitylacetamide): 6b

Yellow solid; Yield: 85%; Rf (chloroform: methanol, 8:2): 0.78; decomposition temperature (^o^C): 183; **FTIR (cm**^**−1**^**)** 3182 (NH), 3031 (sp^2^ CH), 2919 (sp^3^ CH), 1697 (C = O),1597 (C = N),1487,(C = C), ^1^H-NMR ((CD_3_))_2_SO, 300 MHz): δ 10.59 (s,2H, NH), 8.34 (s, 4H, Ar–H), 6.79 (s, 4H, Ar–H), 5.79 (s, 4H, CH_2_), 2.89 (s, 6H, CH_3_), 2.02 (s, 12H, CH_3_); ^13^C-NMR ((CD_3_))_2_SO, 75.5 MHz): δ 167.9 (C = O), 164.1(C = N), 138.7, 136.7, 128.9, 127.7, 122.7 (Ar–C), 54.2, 18.1, 15.1. Anal. Calcd. for C_30_H_32_N_10_O_2_: C, 63.80; H, 5.71; N, 24.81; found: C, 63.85; H, 5.76; N, 24.87. HRMS Caled for C_30_H_32_N_10_O_2_ + H: 564.2710. Found 564.2709.

#### 2,2’-(5,5’-(1,4-phenylene)bis(1H-tetrazole-5,1-diyl))bis-N-(2,3-dichlorophenyl) acetamide: 6c

Pale yellow solid; Yield: 82%; Rf (chloroform: methanol, 8:2): 0.75; decomposition temperature (^o^C): 197; **FTIR (cm**^**−1**^**)** 3174 (NH), 3042 (sp^2^ CH), 2930 (sp^3^ CH), 1697 (C = O),1567 (C = N),1469,(C = C) ^1^H-NMR ((CD_3_))_2_SO, 300 MHz): δ 11.10 (s,2H, NH), 8.31 (s, 4H, Ar–H), 7.38 (s, 2H, Ar–H), 7.61–7.89 (m, 6H, Ar–H), 5.85 (s, 4H, CH_2_); ^13^C-NMR ((CD_3_))_2_SO, 75.5 MHz): δ 168.1, 164.7, 139.4, 138.7, 134.3, 128.2, 127.5, 124.7, 122.4, 119.5, 118.5, 56.7, 21.8. Anal. Calcd. for C_24_H_16_Cl_4_N_10_O_2_: C, 46.62; H, 2.61; N, 22.64; found: C, 46.68; H, 2.65; N, 22.69. HRMS Caled for C_24_H_16_Cl_4_N_10_O_2_ + H: 618.0182. Found 618.0180.

#### 2,2’-(5,5’-(1,4-phenylene)bis(1H-tetrazole-5,1-diyl))bis-N-(2,4-dinitrophenyl) acetamide: 6d

Brown solid; Yield: 75%; Rf (chloroform: methanol, 8:2): 0.65; decomposition temperature (^o^C): 210; **FTIR (cm**^**−1**^**)** 3197 (NH), 3048 (sp^2^ CH), 2944 (sp^3^ CH), 1694 (C = O),1567 (C = N),1479,(C = C) ^1^H-NMR ((CD_3_))_2_SO, 300 MHz): δ 11.63 (s,2H, NH), 9.27 (s, 4H, Ar–H), 8.63 (d, 2H, Ar–H, J = 6.8 Hz), 8. 37 (s, 4H, Ar–H), 7.87 (d, 2H, J = 6.8 Hz), 5.95 (s, 4H, CH_2_); ^13^C-NMR ((CD_3_))_2_SO, 75.5 MHz): δ 169.5, 165.7, 143.4, 138.7, 134.3, 128.2, 127.5, 124.7, 122.4, 119.5,, 56.7. Anal. Calcd. for C_24_H_16_N_14_O_10_: C, 43.64; H, 2.44; N, 29.68; found: C, 43.69; H, 2.49; N, 29.75. HRMS Caled for C_24_H_16_N_14_O_10_ + H: 660.1174. Found 660.1171.

#### 2,2’-(5,5’–(1,4-phenylene)bis(1H-tetrazole-5,1-diyl))bis-N-(2-chlorophenyl) acetamide: 6e

Yellow solid; Yield: 80%; R_f_ (chloroform: methanol, 8:2): 0.77; decomposition temperature (^o^C): 170; **FTIR (cm**^**−1**^**)** 3217 (NH), 3033 (sp^2^ CH), 2940 (sp^3^ CH), 1666 (C = O),1569 (C = N),1499,(C = C) ^1^H-NMR ((CD_3_))_2_SO, 300 MHz): δ 11.10 (s,2H, NH), 8.27 (s, 4H, Ar–H), 7.23–7.79 (m, 8H, Ar–H), 5.95 (s, 4H, CH_2_); ^13^C-NMR ((CD_3_))_2_SO, 75.5 MHz): δ 167.5, 163.9, 139.4, 135.7, 131.7, 128.2, 127.5, 124.7, 122.4, 119.5, 52.7. Anal. Calcd. for C_24_H_18_Cl_2_N_10_O_2_: C, 52.46; H, 3.31; N, 25.50; found: C, 52.44; H, 3.34; N, 25.53. HRMS Caled for C_24_H_18_Cl_2_N_10_O_2_ + H: 548.0991. Found 548.0989.

#### 2,2′-(5,5′- (1,4-phenylene)bis(1H-tetrazole-5,1-diyl))bis-N-(4-chlorophenyl) acetamide: 6f

Yellow solid; Yield: 78%; Rf (chloroform: methanol, 8:2): 0.78; decomposition temperature (^o^C): 170; **FTIR (cm**^**−1**^**)** 3193 (NH), 3031 (sp^2^ CH), 2956 (sp^3^ CH), 1696 (C = O),1598(C = N),1531,(C = C) ^1^H-NMR ((CD_3_))_2_SO, 300 MHz): δ 13.83 (s, 2H, NH), 8.28 (s, 4H, Ar–H), 7.68 (d, 4H, J = 6.9 Hz, Ar–H) 7.24 (d, 4H, J = 6.9 Hz, Ar–H), 5.81 (s, 4H, CH_2_); ^13^C- MR ((CD_3_))_2_SO, 75.5 MHz): δ 165.8 (C = O), 163.1 (C = N), 140.0, 133.6, 129.0, 127.7, 124.2, 119.3 (Ar–C), 61.7. Anal. Calcd. for C_24_H_18_Cl_2_N_10_O_2_: C, 52.46; H, 3.31; N, 25.50; found: C, 52.44; H, 3.34; N, 25.53. found: C, 52.44; H, 3.34; N, 25.53. HRMS Caled for C_24_H_18_Cl_2_N_10_O_2_ + H: 548.0991. Found 548.0989.

### Computational investigations

#### Density functional theory studies

The accurate prediction of the electronic structures of newly synthesized compounds is crucial in drug discovery and development. In this study, we employed a combination of hypothetical and theoretical methods to compute accuracy and economy in molecular docking studies. Specifically, we utilized the density functional theory (DFT) to calculate the electronic structures of the synthesized compounds. To optimize the molecular structures of the compounds, we used the density functional three-parameter hybrid model (DFT/B3LYP) at the 6-311 +  + G(d) basis set level [21, 22]. B3LYP (Becke three-parameter Lee–Yang–Parr) is a hybrid density functional theory (DFT) exchange–correlation functional that incorporates the benefits of Hartree–Fock (HF) and local density approximation (LDA). B3LYP contains a portion of exact exchange, which results in more accurate predictions of bond lengths and angles, as well as electronic properties such as ionization energies and electron affinities. Furthermore, B3LYP is less sensitive to self-interaction errors than pure DFT functionals such as BLYP, BP86, and PBE, resulting in more accurate predictions of reaction energies and reaction pathways [23]. The choice of the basis set is critical in accurately describing the electronic properties of the compounds, while keeping the computational cost reasonable [24]. The Gaussian 09W program [25] package was used to construct the basis set. We validated the accuracy of the DFT/B3LYP calculations by comparing the theoretical results with available experimental data. Additionally, we used theoretical methods to predict the properties of compounds before they were synthesized, saving time and resources. The 6-311 +  + G(d) basis set is a commonly used and well-established basis set that has been shown to provide reliable results for organic molecules. Therefore, we believe that the combination of DFT/B3LYP and the 6-311 +  + G(d) basis set provides an accurate and efficient approach for studying the electronic structures of newly synthesized compounds [26–33]. Finally, the thermodynamic properties were retrieved theoretically from harmonic vibrations as discussed in reported study [34].

#### Molecular docking analysis

Molecular docking was employed to investigate the interactions between newly synthesized compounds and specific target proteins. The 3D crystal structures of TP53, NF-KAPPA-B P65, and caspase-3 were retrieved from the Protein Data Bank (www.rcsb.com, PDB IDs: 3DCY, 1NFI, and 3DEI)[35]. Prior to docking analysis, the proteins were prepared using MGL tools, which involved removing water molecules and heteroatoms, adding Kollman charges and polar hydrogen atoms, and correcting any missing residues [36]. The synthesized compounds were prepared using ChemDraw 3D [37], which included the addition of hydrogen atoms and energy minimization. The compounds were then docked with the target proteins using AutoDock's default genetic algorithm as the scoring function. The grid box dimensions were set as follows: (x; 30.483200, y; 32.901400; z; − 2.936000) for 3DCY, (x: − 8.458537, y: 55.130635, z: − 29.220624) for 1NFI, and (x; − 47.297718, y; 9.871887, z; − 24.212191) for 3DEI. A total of 100 different poses were generated for each protein, and the most stable pose with the lowest energy was selected for further analysis. Finally, the most stable pose was analyzed in 2D and 3D designs to understand the interaction between the compound and the target protein. The findings of this study have the potential to facilitate the design of novel compounds with improved binding affinities to the target proteins.

#### Molecular dynamics simulations

Molecular dynamics (MD) simulation is a powerful computational tool that enables the atomic-level investigation of biomolecular systems [38]. On our system, MD simulations were performed for this study using the Desmond software suite [39].

To begin, we enclosed the system in a 10-angstrom-long-sided orthorhombic rectangular box. The system was infused with water molecules, and counterions were added to reduce the total charge. To characterize the atomic interactions, the OPLS3 force field was utilized [40]. The system underwent a 2000-step energy minimization procedure to eradicate all deleterious interactions and achieve an initial state of stability. During the simulation, we maintained the system's temperature and pressure with a Nose–Hoover thermostat and a Martyna-Tobias-Klein barostat, respectively [41]. Using periodic boundary conditions to represent an infinite system, we ran a 100-ns simulation of production. Root-mean-square-deviation (RMSD), root-mean-square-fluctuation (RMSF), and contact maps were just a few of the metrics utilized to analyze the simulated trajectories. RMSF describes local flexibility, whereas RMSD measures how far the protein backbone structure deviates from its initial state. Using contact mapping, one can learn about protein residues that interact with one another. This MD simulation study made use of an extended orthorhombic rectangular box, the OPLS3 force field, the Nose–Hoover thermostat, and the Martyna-Tobias-Klein barostat, as well as the analysis of root-mean-square deviation (RMSD), root-mean-square force (RMSF), and contact maps to gain insight into the behavior and interactions of the studied system [25].

## Results and discussion

The synthesis of bis-tetrazole acetamides is outlined in Scheme 1. Amides of bis-1,4-(1*H*-tetrazole) was afforded by using 1,4-dicyanobenzene as a starting material. In first step 5-(4-(1*H*-tetrazole-5-yl)phenyl)-1*H*-tetrazole **2** was synthesized by the reaction of 1,4-dicyanobenzene and NaN_3_ in water in the presence of ZnCl_2_ as a catalyst. Compound **3** was treated with ethyl chloro-acetate in potassium carbonate as a base to form diethyl 2,2′-(5,5′-(1,4-phenylene)bis(1*H*-tetrazole-5,1-diyl))diacetate **3.** compound **3** on hydrolysis give a respective acid which on further reaction with thionyl chloride followed by substituted anilines give 2,2′-(5,5′-(1,4-phenylene)bis(1*H*-tetrazole-5,1-diyl))bis-*N*-(substituted phenyl) acetamide **(6a-f)** all of the products were obtained in excellent yield.

Amides of bis-1,4-(1*H*-tetrazole) was characterized by using 1H-NMR and ^13^C-NMR spectroscopy. In ^1^H-NMR spectrum, doublet for a 2 aromatic proton appears at δ 8.34 ppm. In ^13^C-NMR spectrum, carbon atom of a tetrazole ring appear at δ 164.1 ppm while ipso-carbon of phenylene ring appear at δ 128.9 and 4 ortho-carbons at δ 127.7 ppm. The ^1^H-NMR spectral data of compound 6f showed characteristic peak for methylene protons at δ 5.79 ppm. Protons of –CH_2_ and –CH_3_ of ethyl group appear at 2.89–2.02 respectively. In ^13^C-NMR spectrum, carbonyl carbon appear at δ 167.9 ppm, carbon of tetrazole ring appear at 164.1 ppm and methylene carbon bonded to carbonyl group appear at 54.2 ppm.

The 1H-NMR spectral data of compounds **6a-f** showed characteristic peak for methylene protons at δ 5–6 ppm. The appearance of signal at 7–8 ppm value was assigned to aromatic protons. The N–H proton of amide appears at 9–13 ppm. In ^13^C- NMR spectrum, carbonyl carbon of amide appear at δ 160–169 ppm, carbon of tetrazole ring appear at 164.3 ppm and methylene carbon bonded to carbonyl group appear at 54.2 ppm.

### Optimized structures

To find the optimal geometrical structures of compounds for computational analysis, single and isolated molecule in gaseous phase, during theoretical calculations is considered. The templates along with their atomic numbering for the title compounds are shown in Table 1.

**Table 1:**
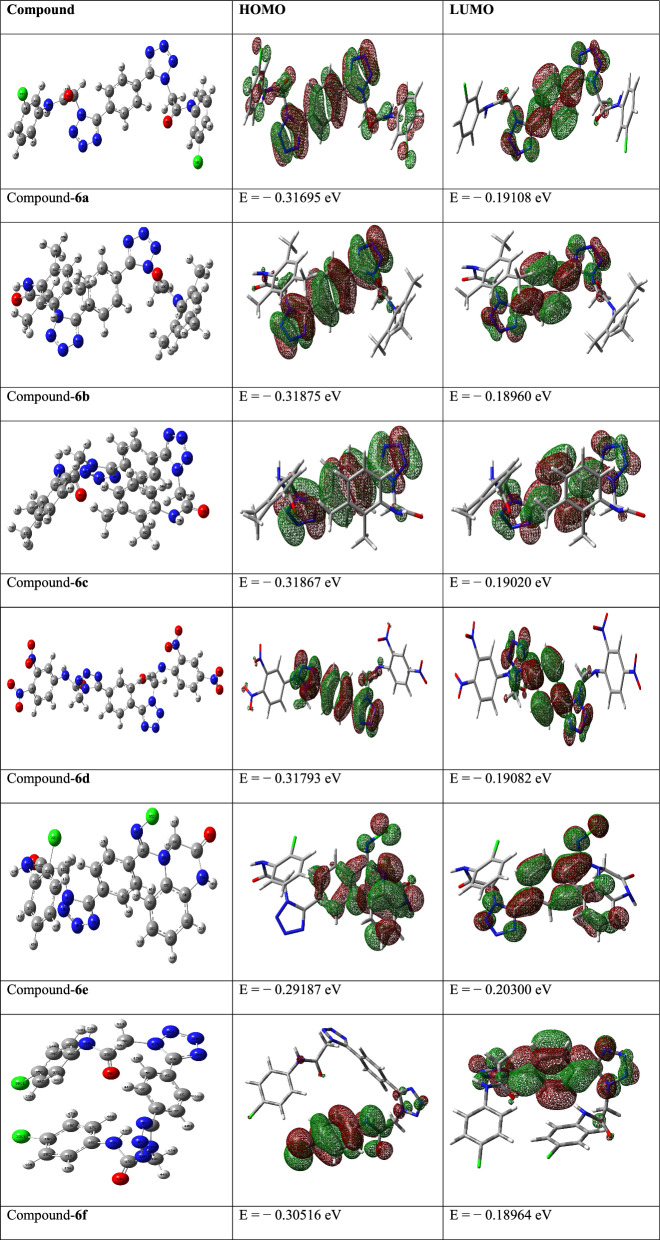
The calculated HOMO–LUMO energies/eV and optimized geometry of the synthesized compounds (B3LYP/6-311 +  + G (d))

### Frontier molecular orbitals (FMO) analysis

While predicting the interactions among molecular orbitals, the highest energy occupied molecular orbital (HOMO) of one molecule and the lowest energy unoccupied molecular orbital (LUMO) of the other molecule is generally considered. The orbital pairs of such interacting molecules exist closest to each in energy of any other pair of orbitals in the two molecules, which allows them to interact efficiently. These orbitals are also named as the frontier orbitals, as they exist at the outermost boundaries of the electrons of the molecules [42, 43]. In finding the molecular electrical transport properties FMOs play crucial role. The energy gap between HOMO and LUMO determines the various physic-chemical characteristics of molecules i.e., kinetic stability, chemical reactivity, polarizability, optical and chemical hardness–softness molecule [44, 45]. The HOMO and LUMO characterizes the ability to give and accept an electron respectively. The energy differences among filled and empty molecular orbitals of **6a-f** considered at B3LYP/6-311 +  + G (d) level is given in Table 1 depicts the chemical reactivity of the molecules.

It is obvious from the Table 1 that the density parameters for the HOMO and LUMO are well defined and the intra-molecular interactions mostly observed inside the ring structure. Usually, the atoms possessing more densities of LUMO should have greater capacity to detach an electron while; the atoms with more occupation of LUMO should have affinity to gain an electron. In case of these bis-tetrazole acetamides, the electronic density resides on the tetrazoles motif and dispersed over the phenyl ring. Compound **6f** possess maximum value of HOMO energy level while other compound **6a-d** and **6e** show uniform variation in the HOMO energy level. Furthermore, compound **6f** also possess minimum LUMO energy value compared to other derivatives. These energy values provided access to extract other important information which is described later in this account.

**Fig. 1:**
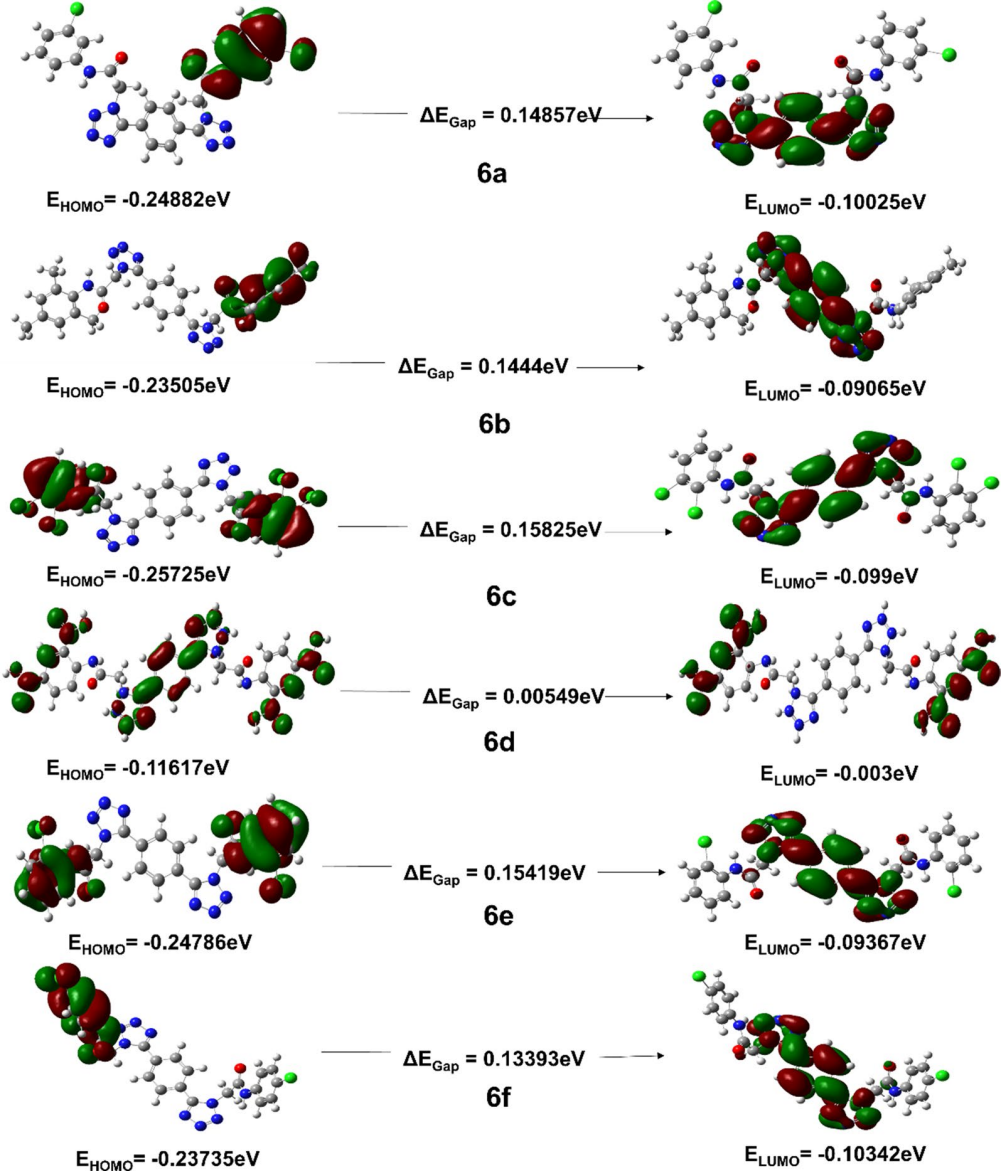
HOMO–LUMO energy analysis conducted at B3LYP/6-31G (d) level of theory

To assess the reliability of the chosen basis set, we conducted a HOMO–LUMO energy analysis of the synthesized derivative using an alternative basis set, namely 6-31G(d). The results obtained from this comparison served to reinforce the accuracy of the selected basis set. Furthermore, by utilizing the B3LYP/6–311 +  + G (d) basis set, we were able to obtain even more precise outcomes than with 6-31G(d). Figure 1 presents the HOMO–LUMO orbitals calculated at the B3LYP/6-31G (d) level of theory, providing visual evidence of the enhanced accuracy achieved through our methodology.

In the present study, we have employed the HOMO LUMO energy values to assess a range of fundamental molecular parameters. Specifically, the parameters of hardness, chemical potential, softness, electronegativity, and electrophilicity index have been evaluated using their respective computational formulas. The hardness parameter, which characterizes the resistance of a molecule to deformation, has been computed as η = 1/2(ELUMO-EHOMO). The chemical potential, which describes the reactivity of a molecule, has been evaluated as µ =—χ. Softness, which is a measure of the response of a molecule to an external perturbation, has been determined using the formula S = 1/2η. Electronegativity, which is indicative of the electron-attracting power of a molecule, has been computed as χ = −1/2(ELUMO + EHOMO). Lastly, the electrophilicity index, which quantifies the electrophilic nature of a molecule, has been determined as ω = µ/2η. It should be noted that the ionization potential (A = −E HOMO) and electron affinity (I = −E LUMO) of the molecule have been used to obtain the aforementioned parameters. In addition, the ionization potential greatly helps to understand, electropositive character, relative reactivity and reducing power of any molecule [46]. All the compounds show subtle variation in the ionization energy as they possess same core motif except the different group attached at phenyl ring Table 1. However, compound **6e** possess ionization potential less than compared to other compounds in the series which indicates the compound **6e** exhibits higher electropositive character, relative chemical reactivity and reducing power.

Electrophilicity index provides close insights regarding structure, reactivity, stability, bonding, toxicity, interactions, and dynamics of the molecule [47]. Moreover it also helps in the analysis of the reactivity outlines in different intra-molecular and intermolecular physicochemical techniques. First time Parr, Szentpaly and Liu provided detail information about the electrophilicity index and now it is employed in the every arena of chemistry [48]. Maynard-Parr modified the concept of electrophilicity index to undermine the diverse class of bio-physicochemical processes [49, 50]. Table 2 summarizes the electrophilicity index value of the newly synthesized derivatives and Compounds **6a-d** and **6f** showed uniform subtle change in the value of electrophilicity index, while compound **6e** did not follow the same pattern and it shows higher value compared to other derivatives of the series. Therefore, compound **6e** can serve as template for designing of interesting tetrazoles containing materials or medicinal agents. The chemical hardness gives clues about the chemical stability. The energy gap between LUMO–HOMO indicates the softness and hardness of the molecule. If the energy gap between two molecules is small or large are termed as soft or hard molecules respectively. Moreover, the hardness and softness of the molecule leads to better understand the polarizability of the molecules. As the energy gap between soft molecules is small hence they can easily be polarized owning to the less amount to energy required for excitation. While hard molecules are hard to be polarized because they required large amount of energy for excitation.

**Table 2:** Comparative Close chemical insight of newly synthesized derivatives (**6a-f**)

Parameters	6a	6b	6c	6d	6e	6f
I (Ionization Potential)	0.3169	0.31875	0.31867	0.31793	0.29187	0.30516
A(Electron Affinity)	0.19108	0.18960	0.19020	0.19082	0.20300	0.18964
E_HOMO_—E_LUMO_	− 0.125915	− 0.12915	− 0.12847	− 0.12711	− 0.08887	− 0.11552
μ (Chemical Potential)	0.254015	0.254175	0.254435	0.254375	0.247435	0.2474
η (Hardness)	0.062935	0.064575	0. 064235	0.063555	0.044435	0.05776
${\varvec{\epsilon}}$(Softness)	7.9447	7.7429	7.7839	7.8672	11.2523	8.6565
Ψ(Electrophilicity index)	0.51262	0.50023	0.50391	0.50906	0.68891	0.52984

The hardness and softness values extracted from the HOMO–LUMO energy levels are summarized in Table 2. The analysis of data given in Table 2, indicated that all the molecules **6a-d** and **6f** showed hardness while compound **6e** showed softness compared to other compounds in the series. In can be inferred from the data of Table 2, that compound **6e** showed softness and hence it can be employed in the designing of charge transfer molecule for electronic devices.

The presented data in Table 3 displays the Fukui indices (f−, f + , and f0) for several atoms of compound 6d (Fukui indices of other compounds is provided in Additional file 1: Table S1 to S6). The f-value indicates the susceptibility of an atom to nucleophilic attacks, while the f + value represents its susceptibility to electrophilic attacks. Finally, f0 is indicative of the atom's intrinsic reactivity.

**Table 3:** Fukui indices for electrophilic and nucleophilic attack for compound **6d**

Atom	Atomic number	f^−^_k_	f + _k_	f0
7	6	0.0001	0	0
8	7	0.016	0	0.008
9	8	0.0001	0	0
10	8	0.9837	0	0.4918
39	6	0	0.0002	0.0001
40	6	0	0.0043	0.0021
41	0	0.5581	0.2791	0.5581
42	0	0.0034	0.0017	0.0034
43	0	0.4335	0.2167	0.4335
44	0	0.0003	0.0001	0.0003
45	0	0.0001	0	0.0001

For atom 7 (carbon), the values of f-k and f + are both 0.0001, indicating that it has no particular susceptibility to either nucleophilic or electrophilic attacks, resulting in an f0 value of 0. For atom 8 (nitrogen), the f- and f + values are 0.016 and 0 respectively, with an f0 value of 0.008, implying that it has a slight tendency to undergo nucleophilic attacks but no particular susceptibility to electrophilic attacks. For atom 9 (oxygen), the values of f-k and f + are both 0.0001, indicating no specific reactivity toward either nucleophiles or electrophiles, thus resulting in an f0 value of 0. For atom 10 (oxygen), the f− and f + values are 0.9837 and 0 respectively, resulting in an f0 value of 0.4918, signifying that it has a high susceptibility to nucleophilic attacks. For atoms 39 and 40 (Carbon in two different environments), the f-k values are both 0, with f + values of 0.0002 and 0.0043 respectively. The resulting f0 values are 0.0001 and 0.0021, indicating that these carbon atoms have little to no reactivity toward nucleophiles but are slightly susceptible to electrophilic attacks. Table 3 is indicating the Fukui indices for compound 6d (Table 2).

**Fig. 2:**
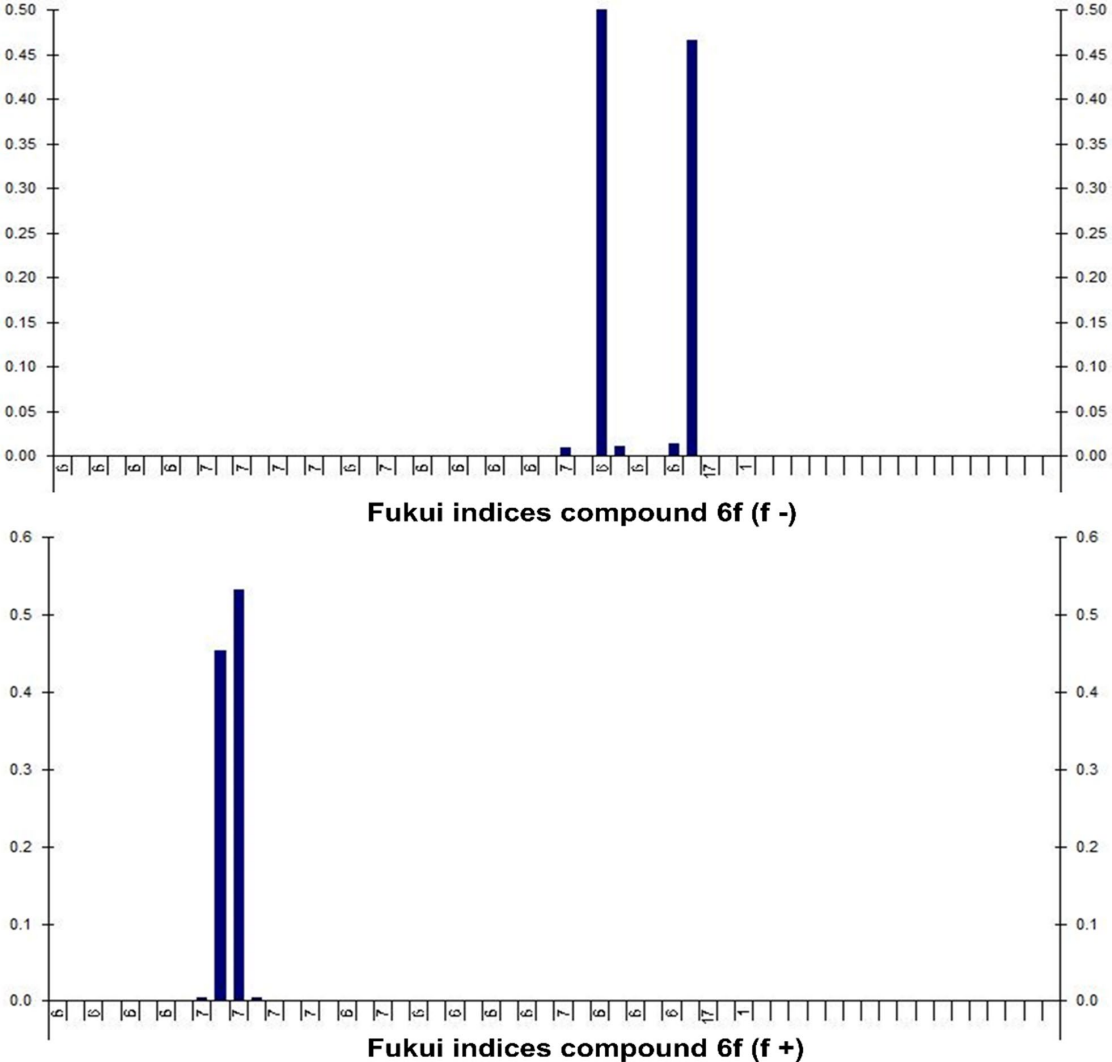
Fukui indices for compound **6f**

The Table 4 depicts the Fukui indices for various atoms in a compound 6f. Fukui indices quantify the reactivity of a molecule's atoms towards electrophilic and nucleophilic attacks. Carbon atoms 1 through 8 and 30–36 are not anticipated to be exceptionally reactive, as their f + k and f-k values are all close to zero. Atoms 9 through 16 are nitrogen atoms, with atom 10 having the highest f + k (0.4526) and f0 (0.2263) values, indicating it is the most reactive to electrophilic and nucleophilic attacks. Atom 31 corresponds to another carbon atom with an exceptionally high f-k value of 0.4997, indicating that it is the most nucleophilic atom in the molecule. The Fig. 2 is illustrating the Fukui indices for compound 6f.

**Table 4:** Fukui indices for electrophilic and nucleophilic attack for compound **6f**

Atom	Atomic number	f^−^_k_	f + _k_	f0
1	6	0	0.0001	0
2	6	0	0.0001	0
3	6	0	0.0005	0.0003
4	6	0	0.0004	0.0002
5	6	0	0.0003	0.0001
6	6	0	0	0
7	6	0	0.0003	0.0001
8	6	0	0	0
9	7	0.0001	0.0039	0.002
10	7	0	0.4526	0.2263
11	7	0	0.5326	0.2663
27	6	0.0001	0.0008	0.0005
28	6	0.0001	0.0005	0.0003
29	7	0.009	0.0009	0.005
30	8	0	0	0
31	6	0.4997	0.0007	0.2502
32	6	0.0112	0.0003	0.0057
33	6	0	0.0002	0.0001
34	6	0	0	0
35	6	0.0138	0	0.0069
36	6	0.4659	0	0.233

Atoms 29 and 30 correspond to nitrogen and oxygen atom respectively, with atom 29 having a comparatively high f + k value of 0.009, indicating that it is more reactive to electrophilic attacks than atom 30. Atoms 32–35 correspond to carbon atoms, with atom 36 having the highest f + k (0.4659) and f0 (0.233) values, indicating it is the most reactive carbon atom towards electrophilic and nucleophilic attacks. The data indicate that atoms 10 and 36 are the most reactive to electrophilic and nucleophilic attacks in the molecule, whereas atoms 1–8 and 30 are predicted to be relatively unreactive.

### Thermodynamic properties

**Fig. 3:**
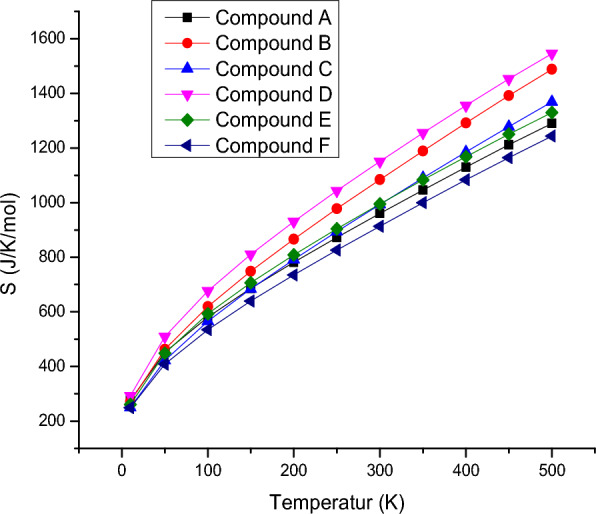
Correlation graphic of entropy and temperature for the bis-tetrazole acetamides **(6a-f)**

**Fig. 4:**
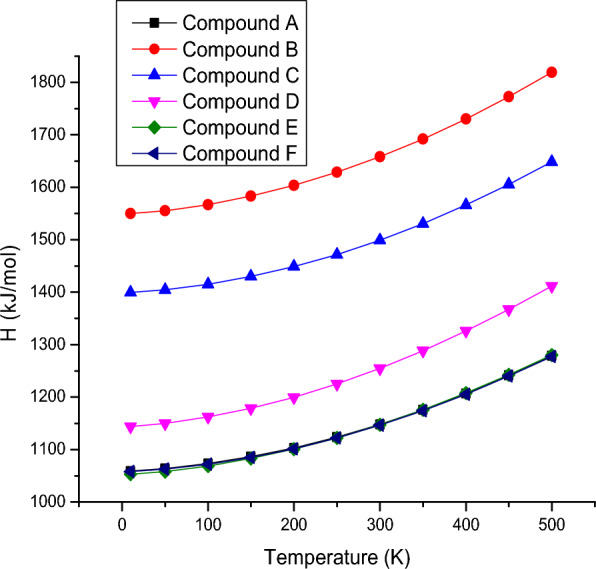
Correlation graphic of temperature and enthalpy for the bis-tetrazole acetamide molecule (**6a-f**)

**Fig. 5:**
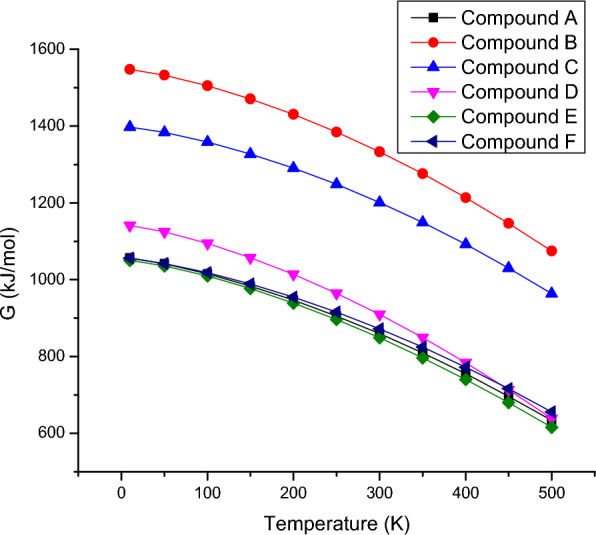
Correlation graphic of temperature and Gibbs free energy for the bis-tetrazole acetamide molecule (**6a-f**)

**Fig. 6:**
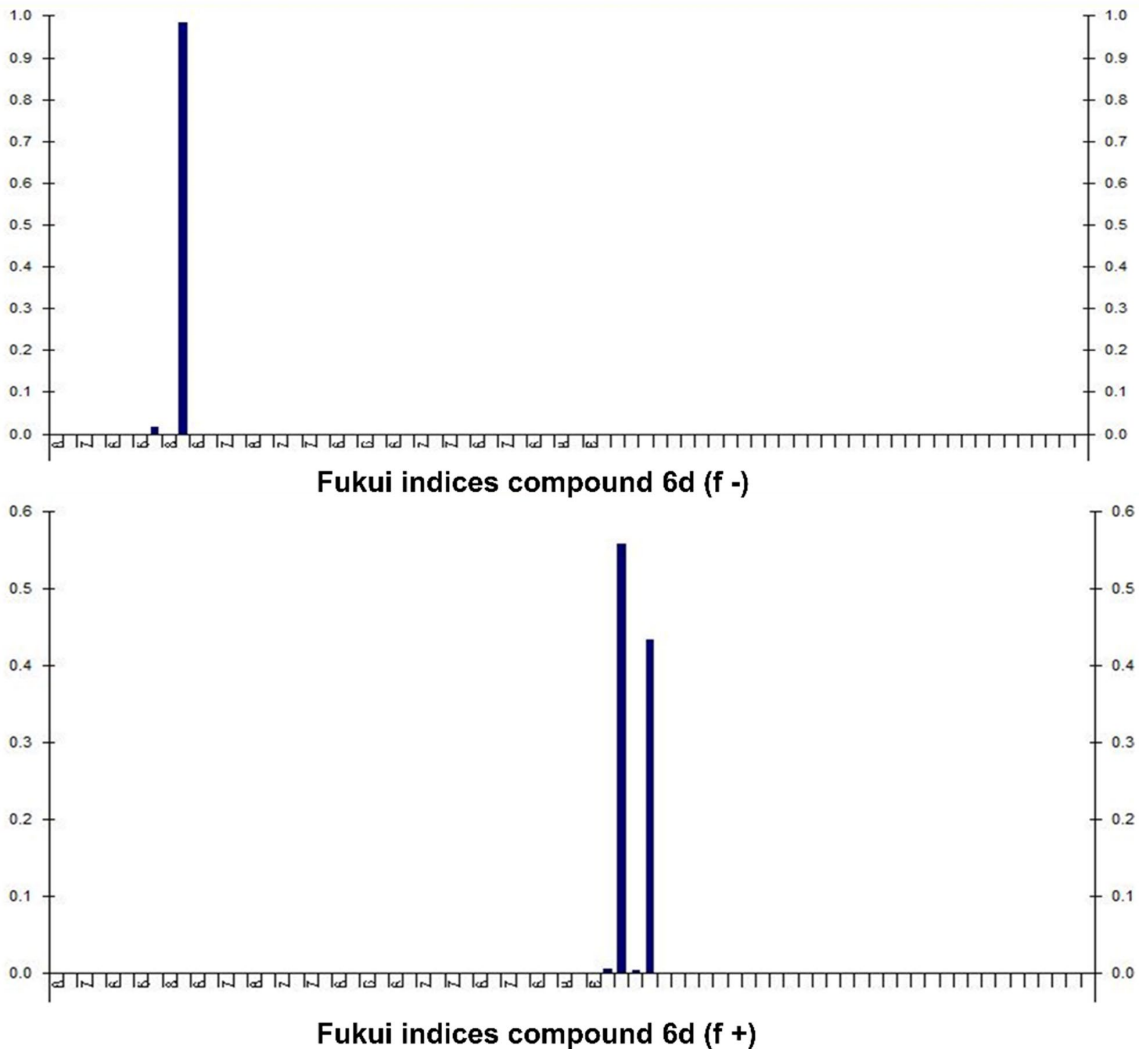
Fukui indices for compound **6d**

The entropies, enthalpies, and Gibbs energy calculated at different temperatures ranging from 10 to 500 K, temperatures and thermodynamic functions correlation equations are given in Figs. 3, 4, 5. It is obvious from the given figures the thermodynamic functions enthalpy, entropy and Gibbs energy of molecular vibration. The entropy of the compound **6f** is minimum compared to other derivatives while compound **6d** possess maximum entropy as is shown in Fig. 1. The correlation of enthalpy with respect to temperature depicted in Fig. 6 shows that compound **6e** and F display almost identical pattern in change of enthalpy with respect to temperature. However, compound **6b** which possesses three methyl units attached with phenyl ring shows maximum enthalpy. In Fig. 2, Gibbs free energy correlation between temperatures reveals that compound **6b** shows maximum energy value at elevated temperature. All the thermodynamic study provides preliminary analysis of fundamental thermodynamic parameters which can provide use informational for detailed studies. According to the second law of thermodynamics and using relations of thermodynamic functions, these basic thermodynamic characteristics enable to predict directions of the chemical reactions. It is significant to observe that all thermodynamic parameters were calculated in gas.

### Oxygen balance and nitrogen percentage

Tetrazoles exhibit large number of nitrogen-nitrogen and carbon–nitrogen bonds as a result have high heat of formation. The tetrazoles have low percentage of C and H bonds and highly insensitive towards electrostatic discharge, impulse or shock. Tetrazoles nucleus is highly desired in energetic materials because it enhances density, keeps good oxygen balance ratio and produce enormous gaseous molecules. Raising nitrogen content by the use of heterocyclic nuclei is reported to improve oxygen balance and forms N_2_ as a product of explosion.

**Fig. 7:**
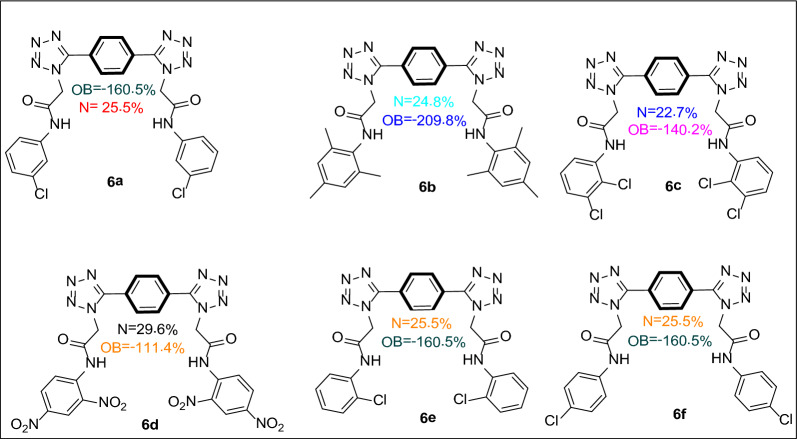
Nitrogen and oxygen balance ratio of synthesized compounds **6a-f**

Tetrazoles are considered as ecofriendly moieties because they possess N–N atoms adjacent to each other which upon decomposition generates N_2_ gas and this makes these unsaturated tetrazoles promising candidates for energetic materials. The oxygen balance and nitrogen percentage were determined by using formula given in the literature. Compound **6d** possess highest percentage of nitrogen and this bears additional nitro groups and serve template for designing of energetic materials. Moreover, the oxygen balance ratio of compound **6d** is minimum compared to other derivatives in the series and this also favor that compound **6d** can possess promising energetic properties as in Fig. 7.

### Molecular docking analysis

Molecular docking is a computer-based technique that enables the investigation of ligand–protein interactions. As a targeting method, molecular docking can be utilized to develop medications that selectively bind to and modify the activity of specific proteins. Caspases 3, kappa, and p53 play crucial roles in apoptosis and cell proliferation, among other processes [51]. Therefore, the therapeutic implications of molecular docking targeting these proteins for the treatment of a variety of diseases, including cancer, are significant. For instance, inhibiting kappa and p53 can reduce tumor development and control cell proliferation, respectively, while inhibiting caspase 3 can induce cancer cell death. The significance of molecular docking-based targeting of caspase 3, kappa, and p53 is highlighted by the potential to develop new and efficacious medications for the treatment of a wide variety of disorders [52]. In this study, we aimed to investigate the binding affinities of synthesized compounds with three distinct proteins, namely TP53, NF-KAPPA-B P65, and caspase-3. To conduct the molecular docking, we obtained the PDB IDs of the target proteins, namely 3DCY (resolution 1.75 Å) for TP53, 1NFI (resolution 2.7 Å) for NF-KAPPA-B P65, and 3DEI (resolution 2.80 Å) for caspase-3, from the Protein Data Bank. Our docking analysis demonstrated a strong interaction between 2,2’-(5,5’-(1,4-phenylene)bis(1*H*-tetrazole-5,1-diyl))bis-N-(2,4-dinitrophenyl) acetamides **(6d)** and TP53 and NF-KAPPA-B P65 with binding energies of -11.8 kJ/mol and -10.9 kJ/mol, respectively. Furthermore, 2,2’-(5,5’–(1,4-phenylene)bis(1*H*-tetrazole-5,1-diyl))bis-N-(2-chlorophenyl) acetamides **(6f)** exhibited potent interaction with caspase-3 with a binding energy of -10.0 kJ/mol as given in Table 5. These findings suggest that the synthesized compounds may have the potential to serve as effective therapeutic agents. The molecular interactions of other synthesized derivatives against respective targeted proteins is provided in (Additional file 1: Table S7). However, to validate their potential, additional experimental studies such as biochemical assays and in-vitro or in-vivo analysis are necessary.

**Table 5:** Molecular docking scores of compounds with targeted protein

PDB ID	Protein	Compound	Docking score kcal/mol	Hydrogen boning residue	Hydrophobic interactions
3DCY	TP53	**6d**	− 11.8	ARG61, HIS 198, GLY199,ARG10, ARG104	ILE22, ARG203, LYS20
1NFI	NF-KAPPA-B P65	**6d**	− 10.9	GLN142, THR164,ASN137, THR136, ARG95, ARG73, VAL163, ARG174, LEU175	GLU92
3DEI	Caspase-3	**6f**	− 10.0	ARG207	HIS121, THR62, ARG64, SER63, SER65, SER209, THR166, SER251, PHE250, PHE256, TYR204, LEU168, TRP206,

These findings suggest that the synthesized compound **6d** can be a potential TP53 inhibitor by targeting the specific amino acid residues in the binding pocket. The formation of hydrogen bonds between **6d** and ARG61, HIS198, GLY199, ARG10, and ARG104 indicates that these residues play an essential role in the binding of the compound to TP53 as shown in Fig. 8. Moreover, the pi-Cation bond between LYS20 and **6d** suggests that this residue may also contribute to the binding affinity of the compound with TP53. Similarly, the binding interactions between the synthesized compounds and NF-KAPPA-B P65 were also investigated. The docking analysis revealed that the amino acid residues ASP68, LYS310, LEU329, GLU333, LEU332, and ARG332 were involved in the binding of the compound with NF-KAPPA-B P65. Among these, ASP68 and LYS310 formed conventional hydrogen bonds with the compound, while LEU329, GLU333, LEU332, and ARG332 formed hydrophobic interactions with the compound. Additionally, the strong interaction between 2,2’-(5,5’-(1,4-phenylene)bis(1H-tetrazole-5,1-diyl))bis-N-(2-chlorophenyl) acetamides and caspase-3 suggests that these compounds may have potential as caspase-3 inhibitors. The binding energy and interactions between the compound and the active site residues of caspase-3 can aid in designing more potent and specific inhibitors for this protein. Overall, the molecular docking results indicate that the synthesized derivatives have potential as therapeutic agents for TP53, NF-KAPPA-B P65, and caspase-3. However, further experimental studies are necessary to validate their activity and selectivity towards these proteins.

**Fig. 8:**
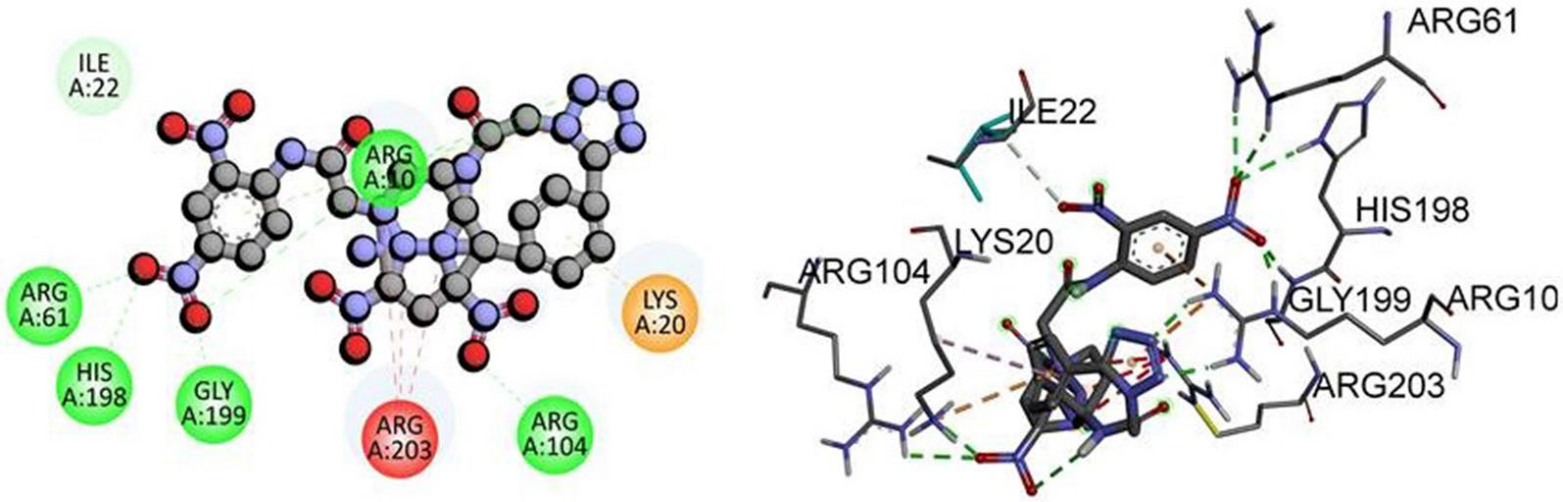
The predicted 2D and 3D binding mode of compound **6d** against TP53

**Fig. 9:**
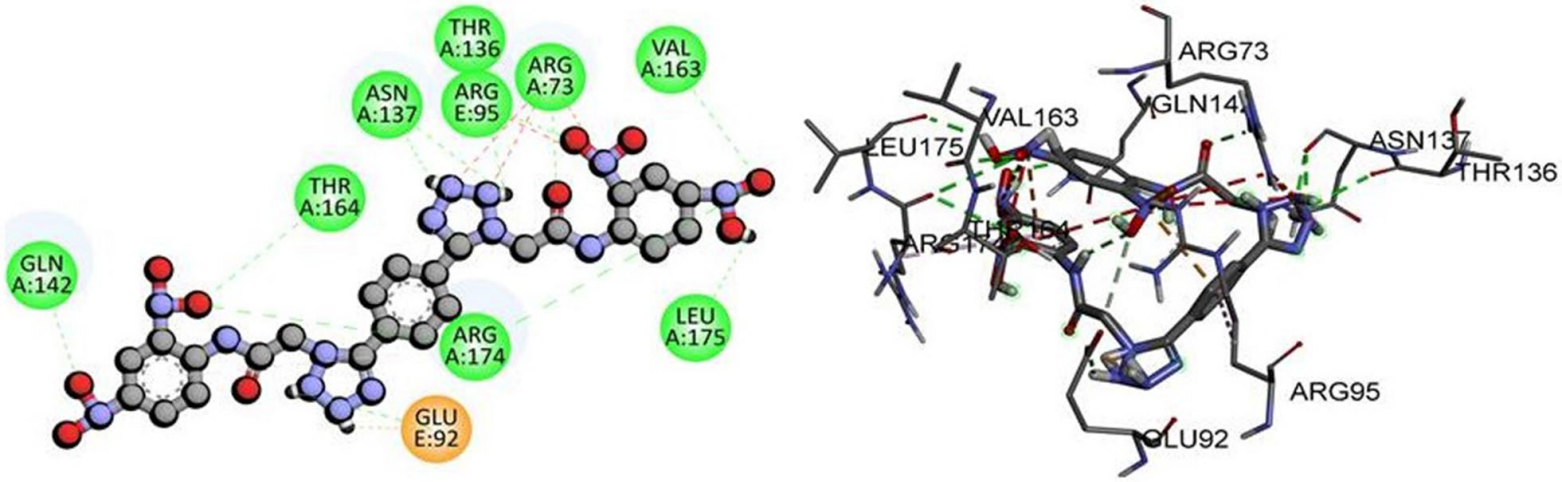
The predicted 2D and 3D binding mode of compound **6d** against NF-KAPPA-B P65

In our study, we performed molecular docking of compound **6d** with the active pocket of NF-KAPPA-B P65 protein. The 2D and 3D binding interactions between 6d and NF-KAPPA-B P65 were analyzed and visualized in Fig. 9. Our docking analysis revealed that several key amino acid residues, including GLN142, THR164, ASN137, THR136, ARG95, ARG73, VAL163, ARG174, and LEU175, formed conventional hydrogen bonds with 6d. Additionally, GLU92 formed a salt bridge with 6d, indicating a strong electrostatic interaction. These results suggest that **6d** may have potential as a therapeutic agent for targeting NF-KAPPA-B P65 protein. However, further experimental studies such as in vitro and in vivo assays are required to confirm the efficacy of **6d** as a therapeutic agent.

**Fig. 10:**
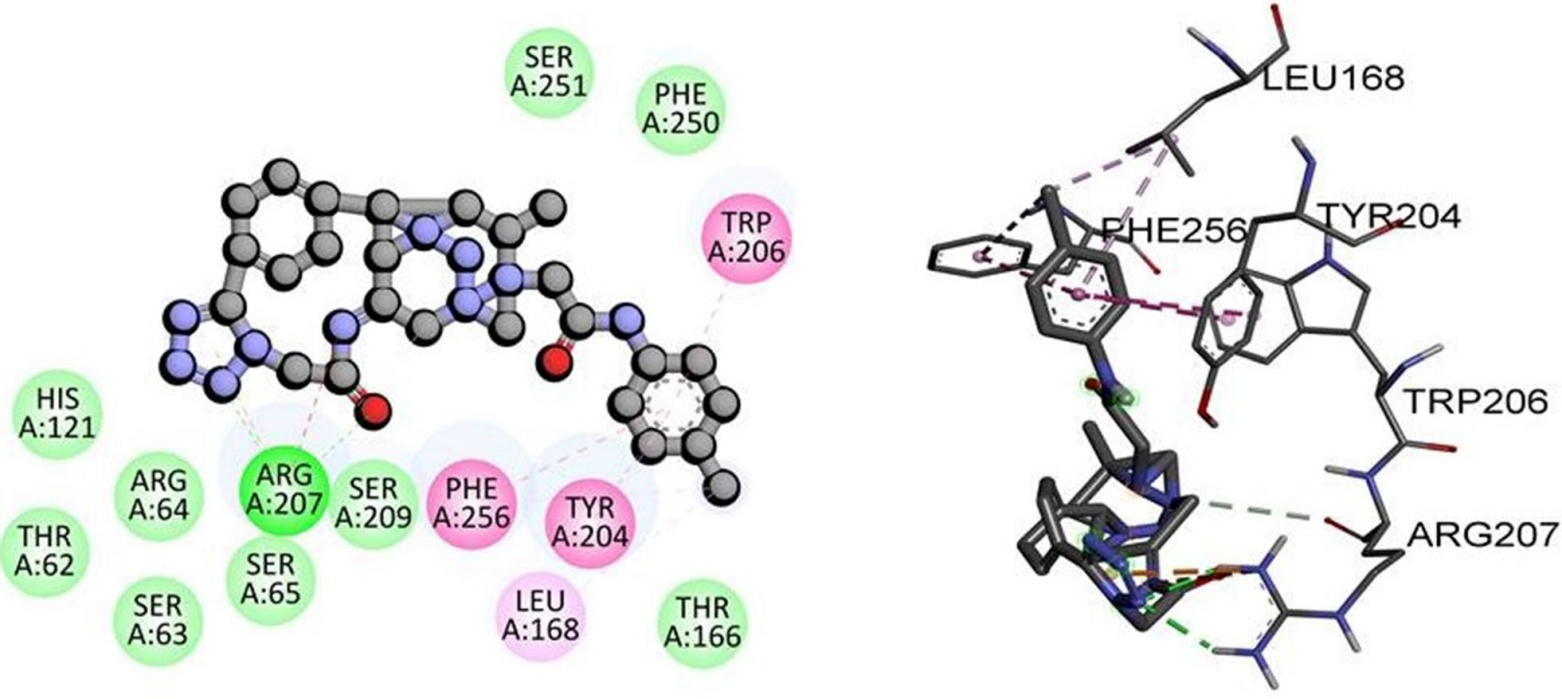
The predicted 2D and 3D binding mode of compound **6f** against caspase-3

In Fig. 7, the 2D and 3D binding interactions between 6f and the active site of caspase-3 are illustrated. The docking analysis revealed that amino acid ARG207 formed a conventional hydrogen bond with **6f**. Additionally, other amino acids including HIS121, THR62, ARG64, SER63, SER65, SER209, THR166, SER251, and PHE250 formed van der Waals interactions with the compound. Furthermore, Leu168 formed alkyl and p-alkyl linkage, while PHE256, TYR204, and TRP206 made pi-pi stacked and pi-pi T-shapes bonding with **6f** compound as shown in Fig. 10. These results indicate that **6f** has the potential to bind strongly with the active site of caspase-3, which could be an effective target for anticancer therapy. However, further experimental studies such as biochemical assays and in-vitro or in-vivo analysis are necessary to confirm the binding affinities and biological activity of these compounds.

### Molecular dynamics simulations

Molecular dynamics (MD) simulations are an outstanding tool for studying the structure and motion of molecules and materials. Protein folding, ligand binding, and molecule-solvent and -surface interactions are just a few of the processes that can be investigated with their aid. The foundation of MD simulations is classical mechanics. By monitoring the locations and velocities of all the atoms in a system over time and applying Newton's equations of motion, the interatomic forces can be determined. This allows the simulation to monitor the system's evolution from its inception to its conclusion.

In the present investigation, MD simulation methodologies were used to assess the stability of the top-ranked conformations against their respective proteins. Notably, compound **6d** exhibited significant molecular interactions with P53 and Kappa protein, while compound **6f** exhibited significant molecular interactions with caspase 3 protein. These best conformations were derived from molecular coupling and subjected to MD simulations to evaluate their stability under simulated conditions. The stability pattern was evaluated using a variety of analytical metrics, including RMSD, RMSF, and contact map analysis (figure given below).

**Fig. 11:**
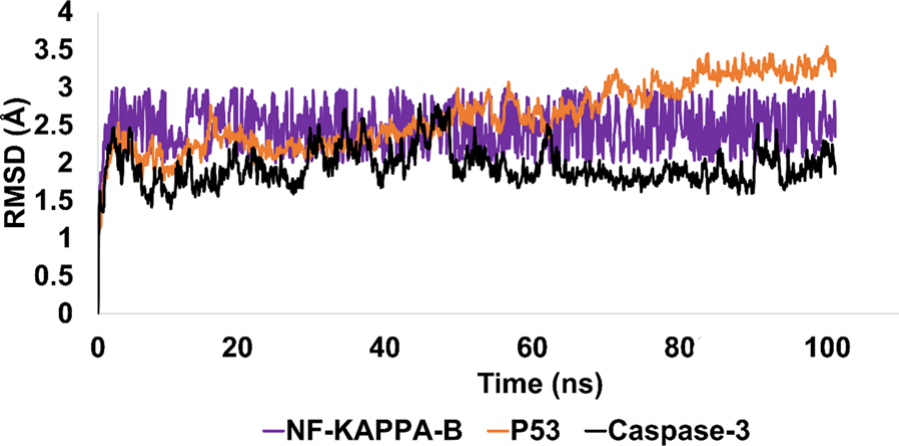
The evolution of RMSD pattern for targeted Apo proteins

RMSD analysis was performed on Apo proteins kappa, P53, and caspase 3 to determine their stability. During the duration of the simulation, none of the three proteins lost their stability. As evidenced by a trajectory that stabilized and balanced at 2.5 angstroms with a mean RMSD of 2.47 angstroms, Kappa protein maintained its structure well throughout the simulation. P53's RMSD increased slightly after 50 ns, but remained well within the permissible range, averaging 2.64 angstroms on average. The inherent flexibility of P53 protein may account for the observed abnormalities. However, apo caspase 3, with an average RMSD of 1.94 angstroms, was the most stable of the three proteins. The protein's structure was maintained within an appropriate RMSD range despite certain fluctuations throughout the simulation. The root-mean-square-deviation (RMSD) analysis reveals that the three apo proteins were stable during the simulation period, with RMSD values within the permissible range. The Fig. 11 is illustrating the evolution of RMSD for all Apo proteins.

RMSD was also utilized to evaluate the stability pattern of liganded proteins. Notable was the fact that 6d exhibited significant binding affinity for Kappa and P53 protein. In contrast, in-silico research revealed that compound 6f was a potent inhibitor of caspase 3. Interpretation of RMSD evolution revealed that the NF-KAPPA-6d complex exhibited initial perturbations lasting 45 ns, after which the perturbations became stable and the trajectory equilibrated. Taking images at various intervals of the simulated trajectory revealed the rearrangements of ligand within the active pocket, which were the initial perturbations. Throughout rearrangements, compound 6d formed strong hydrophobic and hydrophilic contacts with active site residues, which stabilized the trajectory. The average RMSD for the simulated complex was 2.40 angstroms, which is an acceptable value.

Regarding the P53-6d complex, it was noteworthy that this complex exhibited significant stability and equilibrium throughout the simulated trajectory. The average RMSD of P53-6d complex was 2.5 angstroms, which is acceptable. After initial rearrangements, ligand developed molecular interactions that persisted throughout the majority of the simulated trajectory. Additionally, ligand remained bound to active site residues, namely Glu255, GLU89, and ALA200.

**Fig. 12:**
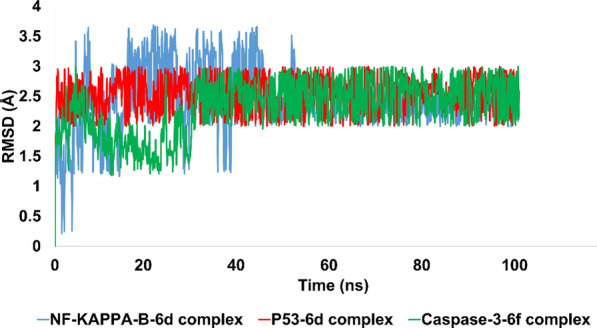
The demonstration of stability pattern for liganded protein using RMSD metric

RMSD analysis of the caspase 3-6f complex revealed the stability and conformational alterations of the complex during simulation (Fig. 12). The complex's initial simulated structure exhibited an RMSD of approximately 1.5 angstroms, which could be attributed to minor structural deviations resulting from the docking procedure. However, minor rearrangements of the ligands in the complex led to the formation of new and stronger contacts with the protein. The establishment of these new contacts resulted in the stabilization and preservation of the complex's trajectory throughout the simulation period. The complex's average RMSD value was determined to be 2.3 angstroms, indicating that it maintained its structural integrity throughout the simulation. This value falls within an acceptable range, suggesting that, despite minor fluctuations during simulation, the complex retained its essential structural characteristics. Overall, the RMSD analysis emphasizes the stability and robustness of the caspase 3-6f complex, as well as the sensitivity of ligand–protein interactions to minor structural alterations.

RMSF analysis is an essential method for determining the structural plasticity and stability of proteins during molecular simulations. It aids in our understanding of the scope of atomic oscillations and sheds light on the dynamic behavior of protein structures.

RMSF analysis provided insights into the structural flexibility and stability of the simulated Kappa, P53, and caspase 3 proteins. The simulation revealed that the Kappa protein is quite flexible, with an average RMSF of 4.4 angstroms. Nonetheless, the ligand-contacting amino acid residues remained relatively stable, indicating that ligand binding had little effect on the overall plasticity of the protein. Throughout the simulation, the P53 protein structure remained comparatively stable, with an average RMSF of 1.8 angstroms. Since the liganded residues were also stable, it appears that ligand binding did not cause significant conformational changes in the protein. Caspase 3, with an average RMSF of 1.4 angstroms, was the most stable of the three examined proteins. Since the RMSF is so minuscule, the protein structure must have remained extremely stable throughout the simulation.

**Fig. 13:**
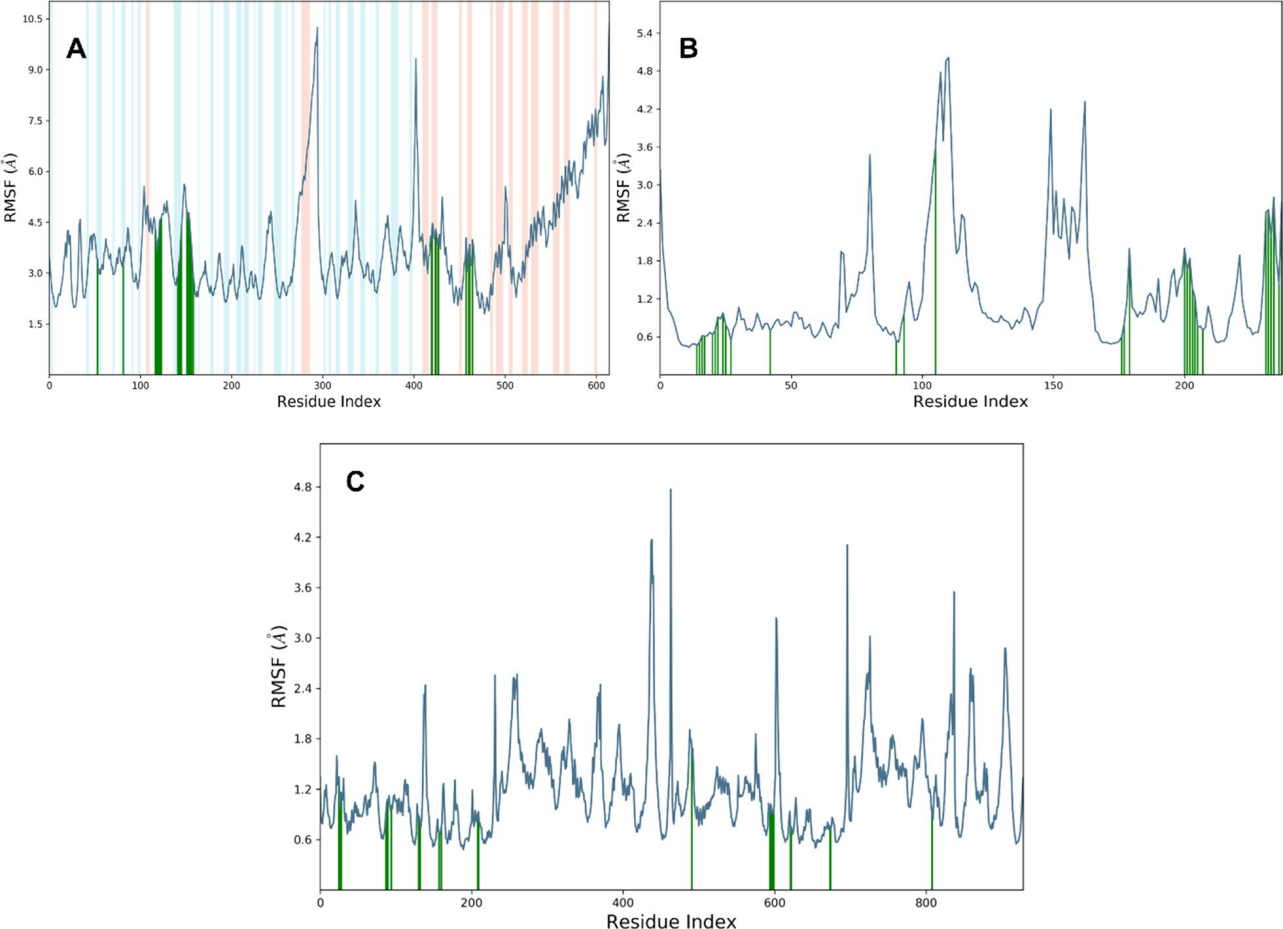
RMSF analysis for each amino acid residue of targeted protein. **A** RMSF for Kappa protein **B** RMSF for P53 **C** RMSF for caspase 3

The C and N terminal residues of the proteins exhibited greater variation, suggesting that these regions are more flexible and dynamic than the protein structures' core. Previous research has demonstrated the importance of these regions for protein folding, stability, and function; therefore, our discovery is consistent with this. The RMSF analysis demonstrates that the protein structures were quite stable after ligand binding and that the structures were maintained throughout the simulation. The relatively low RMSF values of P53 and caspase 3 provide additional evidence of their high stability and rigidity, which may have implications for their biological function and therapeutic targeting. Figure 13 is illustrating the RMSF analysis for each targeted proteins.

**Fig. 14:**
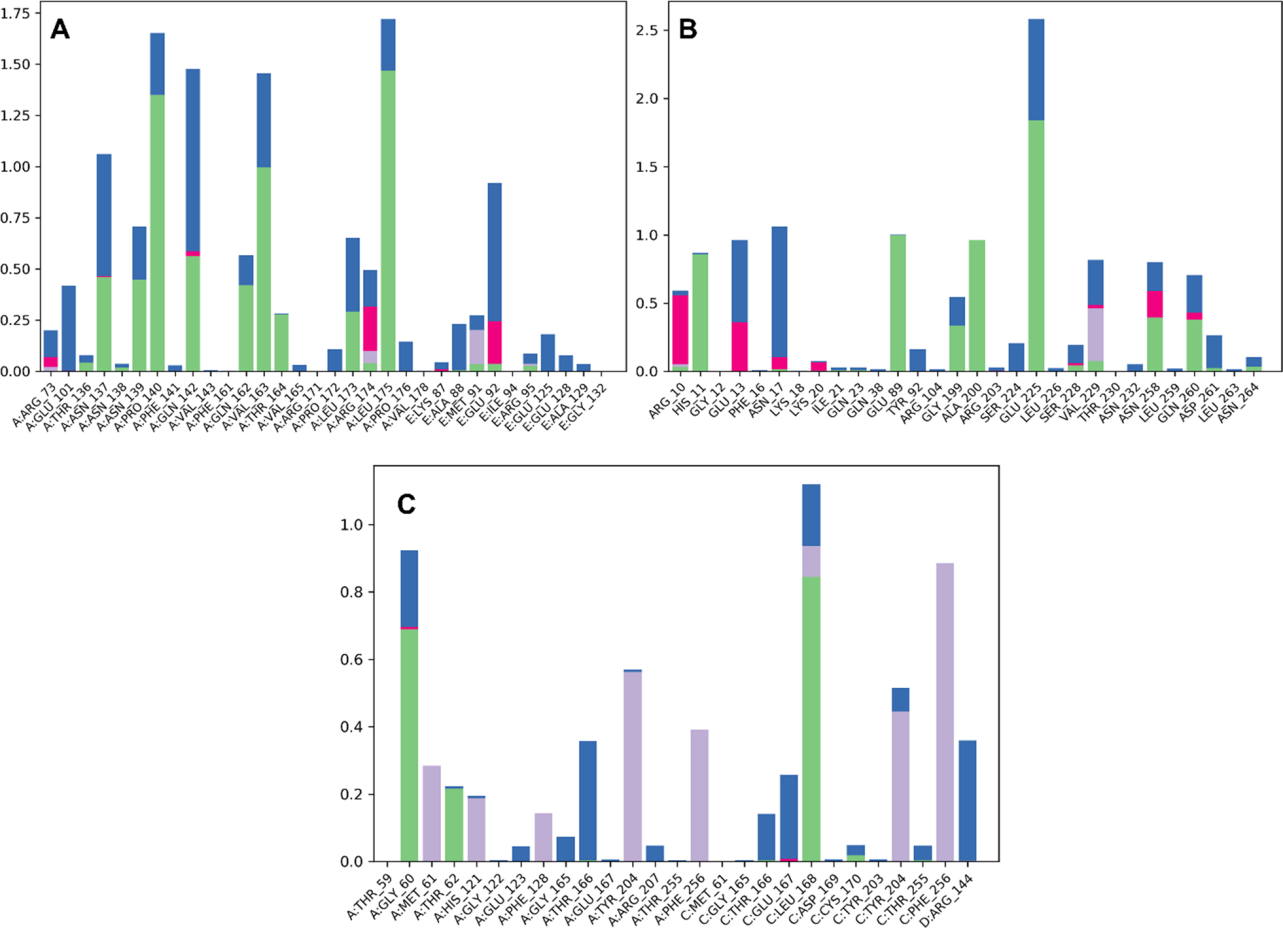
Contact map analysis **A** Kappa-6d complex **B** P53-6d complex **C** Caspase 3-6f complex

Contact map analysis provides a mechanistic understanding of the intensity of molecular interactions. The contact map interpretation demonstrated that stronger interactions were formed with the targeted proteins. Specifically, compound 6d interacted with essential amino acid residues of kappa and P53 proteins. ASN137, ASN139, PRO140, GLN142, GLN162, VAL163, THR164, LEU173, AND LEU175 of the Kappa protein were observed to be involved in hydrogen bonding. Interaction fractions ranging from 50 to 150% of the simulated trajectory represented the strength of hydrogen bonding interactions. In addition, water bridges, cationic and hydrophobic interactions contributed to the protein ligand's stability. In contrast, compound 6d also interacted with essential P53 protein residues. Specifically, GLU89, GLY199, ALA200, GLU225, GLN260, and ASN258 were involved in hydrogen bonding with interaction fractions ranging from 50 to 175 percent of the simulated trajectory. Additionally, ARG10, GLU13, and ASN17 were involved in cationic interactions with 6d. Additionally, water bridges and hydrophobic interactions stabilized the protein–ligand complex. Finally, the mechanistic insight revealed that compound 6f interacted hydrophobically and hydrophilically with caspase 3's essential residues. GLY60, THR62, and LEU168 were heavily involved in hydrogen bonding, with interaction fractions exceeding 60%, 20%, and 80%, respectively. In addition, hydrophobic interactions with MET61, PHE128, TYR204, PHE256, TYR204, and PHE256 were observed. All of these interactions contributed to the stabilization of the protein–ligand complex. Figure 14 depicts the comprehensive contact map analysis for each complex.

### Quantitative structure activity relationship

In the current study, we have synthesized and characterized a novel series of 2,2’-(5,5’-(1,4-phenylene)bis(1H-tetrazole-5,1-diyl))bis-N-acetamides derivatives. We found that the activity of the compounds is correlated to the substitution pattern on the phenyl rings. Specifically, we related the in-silico binding affinity of the compounds to the different substituents present on the phenyl rings.

Our results showed that compounds 6d and 6f exhibited significant inhibitory potential against the targeted proteins. The dinitro and para chloro substitution at the terminal benzene rings of compound 6d and 6f, respectively, played a crucial role in enhancing the binding affinity of the compounds to the target proteins. Compound 6d, bearing the dinitro substitution, established strong hydrogen bonding interactions with important amino acid residues in the active site of TP53 and NF-KAPPA-B P65, resulting in strong binding energy. We also found that the position of the substituent on the phenyl ring is crucial in establishing the inhibitory potential of the compound. Ortho and meta substituted chloro groups decreased the inhibitory potential of the compounds, as observed in compounds 6e, 6a, and 6c, respectively. Interestingly, we observed that unsubstituted terminal benzene rings in compound 6b demonstrated the least inhibitory activity against the targeted proteins. This indicates the importance of appropriate substitutions on the phenyl rings for achieving desired activity. In summary, our study highlights the significance of the structure–activity relationship (SAR) in designing novel compounds with potent inhibitory activity against targeted proteins. The SAR analysis of our compounds showed that appropriate substitutions on the phenyl rings are crucial for enhancing the binding affinity and inhibitory potential of the compounds.

## Conclusion

A new series of 2,2’-(5,5’-(1,4-phenylene)bis(1H-tetrazole-5,1-diyl))bis-N-acetamides based on a Terephthalonitrile have been successfully synthesized structures of a synthesized molecules have been characterized on the basis of ^1^H-NMR and ^13^C-NMR. Spectral figures showing excellent correlations with the structure of molecules. Similarly, synthesis of amide of bis-1,4-(*1H*-tetrazole) carried out starting from 1,4-dicyanobenzen in four steps. All of the products are obtained in very good yield. Density functional theory (B3LYP/6-311G +  + (d)) considerations revealed the substantial chemical reactivity potential as indicated by Fukui indices and global reactivity parameters. In addition, molecular docking studies revealed significant interactions of synthesized derivatives against cancer proteins, which suggest possible anti-cancer potential of these heterocyclic compounds. The molecular docking studies were further strengthen by 100 ns MD simulations which revealed that protein–ligand complex remained stable throughout the simulated trajectory suggesting these derivatives as potential inhibitors of cancer proteins.

The outcomes help out in the mission of experimental and theoretical proof for the title compounds as reaction intermediates for aryl coupling reactions such as Suzuki coupling, and provide basic framework for the designing of energetic materials.

### Supplementary Information

**Additional file 1. Table S1.** FUKUI indices for compound 6a. **Table S2.** FUKUI indices for compound 6b. **Table S3.** FUKUI indices for compound 6c. **Table S4.** FUKUI indices for compound 6d. **Table S5.** FUKUI indices for compound 6e. **Table S6.** FUKUI indices for compound 6f. **Table S7.** Molecular interactions of synthesized derivatives against targeted protein. **Figure S1.** FTIR Spectra of Compound 6b. **Figure S2.** 1H NMR Spectra of Compound 6b. **Figure S3.** 13C NMR Spectra of Compound 6b. **Figure S4.** FTIR Spectra of Compound 6f. **Figure S5.** 1H NMR Spectra of Compound 6f. **Figure S6.** 13C NMR Spectra of Compound 6f.

## Data Availability

Data will be available on request by the corresponding author.
