# Supplementary material for: Computational and theoretical chemistry of newly synthesized and characterized 2,2’-(5,5’-(1,4-phenylene)bis(1H-tetrazole-5,1-diyl))bis-N-acetamides
Source: BMC Chem. 2023 Aug 14;17(1):97. doi: 10.1186/s13065-023-01011-3 (PMC10426183; doi:10.1186/s13065-023-01011-3)
Supplement: Supplementary file 1 — Additional file 1. Table S1. FUKUI indices for compound 6a. Table S2. FUKUI indices for compound 6b. Table S3. FUKUI indices for compound 6c. Table S4. FUKUI indices for compound 6d. Table S5. FUKUI indices for compound 6e. Table S6. FUKUI indices for compound 6f. Table S7. Molecular interactions of synthesized derivatives against targeted protein. Figure S1. FTIR Spectra of Compound 6b. Figure S2. 1H NMR Spectra of Compound 6b. Figure S3. 13C NMR Spectra of Compound 6b. Figure S4. FTIR Spectra of Compound 6f. Figure S5. 1H NMR Spectra of Compound 6f. Figure S6. 13C NMR Spectra of Compound 6f. [file 13065_2023_1011_MOESM1_ESM.docx]

**Computational and theoretical chemistry of newly Synthesized and characterized 2,2^’^-(5,5^’^-(1,4-phenylene)bis(1*H*-tetrazole-5,1-diyl))bis-*N*-acetamides**

Syeda Abida Ejaz^a^*, Aftab Farid^b^, Seema Zargar^c^, Pervaiz Ali Channar^d^, Mubashir Aziz^a^, Tanveer A. Wani^c^, Hafiz Muhammad Attaullah^b^, Rabail Ujhan^e^, Arfa Tehzeeb^f^, Aamer Saeed^b^*, Hafiz Saqib Ali^g,h^, Mauricio F. Erben^i^

*^a^Department of Pharmaceutical Chemistry, Faculty of Pharmacy, The Islamia University of Bahawalpur, Bahawalpur 63100, Pakistan*

*^b^Department of Chemistry, Quaid-i-Azam University Islamabad, Islamabad 45320, Pakistan*

*^c^Department of Pharmaceutical Chemistry, College of Pharmacy, King Saud University, P.O. Box 2457,Riyadh 11451, Saudi Arabia*

*^d^Department of Basic Sciences and Humanities, Faculty of of Information Science and Humanities, Dawood University of Engineering and Technology Karachi, Karachi 74800, Pakistan*

*^e^Dr. M. A. Kazi Institute of Chemistry, University of Sindh, Jamshoro 76080. Pakistan*

*^f^Department of Pharmacy, Quaid-i-Azam University Islamabad, Islamabad 45320, Pakistan*

*^g^Manchester Institute of Biotechnology, The University of Manchester, 131 Princess St, Manchester, M1 7DN, United Kingdom,*

*^h^School of Chemistry, The University of Manchester, Oxford Road, Manchester, M13 9PL, United Kingdom*

*^i^CEQUINOR (UNLP, CONICET-CCT La Plata), Departamento de Química, Facultad de Ciencias Exactas, Universidad Nacional de La Plata, C.C. 962 (1900), La Plata, República Argentina*

***Corresponding Authors:**

Aamer Saeed, Department of Chemistry Quaid-i-Azam University-45320, Islamabad, Pakistan*.*  aamersaeed@yahoo.com. Syeda Abida Ejaz, Department of Pharmaceutical Chemistry, Faculty of Pharmacy, The Islamia University of Bahawalpur, Bahawalpur 63100, Pakistan. abida.ejaz@iub.edu.pk

**Additional file 1**

Table S1. FUKUI indices for compound 6a

| **N** | **Z** | **f-** | **f+** | **f0** |
| --- | --- | --- | --- | --- |
| 1 | 6 | 0 | 0 | 0 |
| 2 | 6 | 0 | 0 | 0 |
| 3 | 6 | 0 | 0 | 0 |
| 4 | 6 | 0 | 0.0002 | 0.0001 |
| 5 | 6 | 0 | 0.0003 | 0.0001 |
| 6 | 6 | 0 | 0.0003 | 0.0002 |
| 7 | 6 | 0 | 0 | 0 |
| 8 | 6 | 0 | 0.0001 | 0.0001 |
| 9 | 7 | 0 | 0 | 0 |
| 10 | 7 | 0 | 0 | 0 |
| 11 | 7 | 0 | 0 | 0 |
| 12 | 7 | 0 | 0 | 0 |
| 13 | 7 | 0 | 0.0045 | 0.0023 |
| 14 | 7 | 0 | 0.5223 | 0.2611 |
| 15 | 7 | 0 | 0.4654 | 0.2327 |
| 16 | 7 | 0 | 0.004 | 0.002 |
| 17 | 6 | 0 | 0.0002 | 0.0001 |
| 18 | 6 | 0 | 0.0002 | 0.0001 |
| 19 | 7 | 0 | 0.0007 | 0.0003 |
| 20 | 8 | 0 | 0 | 0 |
| 21 | 6 | 0 | 0.0006 | 0.0003 |
| 22 | 6 | 0 | 0.0003 | 0.0001 |
| 23 | 6 | 0 | 0.0002 | 0.0001 |
| 24 | 6 | 0 | 0 | 0 |
| 25 | 6 | 0 | 0 | 0 |
| 26 | 6 | 0 | 0 | 0 |
| 27 | 6 | 0 | 0 | 0 |
| 28 | 6 | 0 | 0 | 0 |
| 29 | 7 | 0 | 0 | 0 |
| 30 | 8 | 0 | 0 | 0 |
| 31 | 6 | 0 | 0 | 0 |
| 32 | 6 | 0 | 0 | 0 |
| 33 | 6 | 0.016 | 0 | 0.008 |
| 34 | 6 | 0.504 | 0 | 0.252 |
| 35 | 6 | 0.4671 | 0 | 0.2335 |
| 36 | 6 | 0.0128 | 0 | 0.0064 |
| 37 | 17 | 0 | 0 | 0 |
| 38 | 17 | 0 | 0 | 0 |
| 39 | 1 | 0 | 0 | 0 |
| 40 | 0 | 0 | 0 | 0 |
| 41 | 0 | 0 | 0 | 0 |
| 42 | 0 | 0 | 0 | 0 |
| 43 | 0 | 0 | 0 | 0 |
| 44 | 0 | 0 | 0 | 0 |
| 45 | 0 | 0.0005 | 0.0002 | 0.0005 |
| 46 | 0 | 0 | 0 | 0 |
| 47 | 0 | 0 | 0 | 0 |
| 48 | 0 | 0 | 0 | 0 |
| 49 | 0 | 0 | 0 | 0 |
| 50 | 0 | 0 | 0 | 0 |
| 51 | 0 | 0 | 0 | 0 |
| 52 | 0 | 0 | 0 | 0 |
| 53 | 0 | 0 | 0 | 0 |
| 54 | 0 | 0 | 0 | 0 |
| 55 | 0 | 0 | 0 | 0 |
| 56 | 0 | 0 | 0 | 0 |

Table S2. FUKUI indices for compound 6b

| **N** | **Z** | **f-** | **f+** | **f0** |
| --- | --- | --- | --- | --- |
| 1 | 6 | 0 | 0 | 0 |
| 2 | 6 | 0 | 0 | 0 |
| 3 | 6 | 0 | 0.0004 | 0.0002 |
| 4 | 6 | 0 | 0.0003 | 0.0001 |
| 5 | 6 | 0 | 0.0002 | 0.0001 |
| 6 | 6 | 0 | 0 | 0 |
| 7 | 6 | 0 | 0.0001 | 0.0001 |
| 8 | 6 | 0 | 0 | 0 |
| 9 | 7 | 0 | 0.0041 | 0.0021 |
| 10 | 7 | 0 | 0.4688 | 0.2344 |
| 11 | 7 | 0 | 0.52 | 0.26 |
| 12 | 7 | 0 | 0.0045 | 0.0023 |
| 13 | 7 | 0 | 0 | 0 |
| 14 | 7 | 0 | 0 | 0 |
| 15 | 7 | 0 | 0 | 0 |
| 16 | 7 | 0 | 0 | 0 |
| 17 | 6 | 0 | 0 | 0 |
| 18 | 6 | 0 | 0 | 0 |
| 19 | 7 | 0 | 0 | 0 |
| 20 | 8 | 0 | 0 | 0 |
| 21 | 6 | 0 | 0 | 0 |
| 22 | 6 | 0 | 0 | 0 |
| 23 | 6 | 0 | 0 | 0 |
| 24 | 6 | 0 | 0 | 0 |
| 25 | 6 | 0 | 0 | 0 |
| 26 | 6 | 0 | 0 | 0 |
| 27 | 6 | 0 | 0.0002 | 0.0001 |
| 28 | 6 | 0 | 0.0002 | 0.0001 |
| 29 | 7 | 0 | 0.0003 | 0.0002 |
| 30 | 8 | 0 | 0 | 0 |
| 31 | 6 | 0 | 0.0002 | 0.0001 |
| 32 | 6 | 0 | 0.0001 | 0 |
| 33 | 6 | 0.0112 | 0 | 0.0056 |
| 34 | 6 | 0.4525 | 0 | 0.2262 |
| 35 | 6 | 0.5142 | 0 | 0.2571 |
| 36 | 6 | 0.0155 | 0.0001 | 0.0078 |
| 37 | 6 | 0 | 0 | 0 |
| 38 | 6 | 0 | 0 | 0 |
| 39 | 6 | 0 | 0 | 0 |
| 40 | 6 | 0 | 0 | 0 |
| 41 | 0.0001 | 0 | 0 | -0.0001 |
| 42 | 0.0065 | 0 | 0.0033 | -0.0065 |
| 43 | 0 | 0 | 0 | 0 |
| 44 | 0 | 0 | 0 | 0 |
| 45 | 0 | 0 | 0 | 0 |
| 46 | 0 | 0 | 0 | 0 |
| 47 | 0 | 0 | 0 | 0 |
| 48 | 0 | 0 | 0 | 0 |
| 49 | 0 | 0 | 0 | 0 |
| 50 | 0 | 0 | 0 | 0 |
| 51 | 0 | 0 | 0 | 0 |
| 52 | 0 | 0 | 0 | 0 |
| 53 | 0 | 0 | 0 | 0 |
| 54 | 0 | 0.0003 | 0.0002 | 0.0003 |
| 55 | 0 | 0 | 0 | 0 |
| 56 | 0 | 0 | 0 | 0 |
| 57 | 0 | 0 | 0 | 0 |
| 58 | 0 | 0 | 0 | 0 |
| 59 | 0 | 0 | 0 | 0 |
| 60 | 0 | 0 | 0 | 0 |
| 61 | 0 | 0 | 0 | 0 |
| 62 | 0 | 0 | 0 | 0 |
| 63 | 0 | 0 | 0 | 0 |
| 64 | 0 | 0 | 0 | 0 |
| 65 | 0 | 0 | 0 | 0 |
| 66 | 0 | 0 | 0 | 0 |
| 67 | 0 | 0 | 0 | 0 |
| 68 | 0 | 0 | 0 | 0 |
| 69 | 0 | 0 | 0 | 0 |
| 70 | 0 | 0 | 0 | 0 |
| 71 | 0 | 0 | 0 | 0 |
| 72 | 0 | 0 | 0 | 0 |
| 73 | 0 | 0 | 0 | 0 |
| 74 | 0 | 0 | 0 | 0 |

Table S3.. FUKUI indices for compound 6c

| **N** | **Z** | **f-** | **f+** | **f0** |
| --- | --- | --- | --- | --- |
| 1 | 6 | 0 | 0 | 0 |
| 2 | 6 | 0 | 0 | 0 |
| 3 | 6 | 0 | 0.0003 | 0.0001 |
| 4 | 6 | 0 | 0.0003 | 0.0001 |
| 5 | 6 | 0 | 0.0002 | 0.0001 |
| 6 | 6 | 0 | 0 | 0 |
| 7 | 6 | 0 | 0.0001 | 0 |
| 8 | 6 | 0 | 0 | 0 |
| 9 | 7 | 0 | 0.004 | 0.002 |
| 10 | 7 | 0 | 0.468 | 0.234 |
| 11 | 7 | 0 | 0.5205 | 0.2602 |
| 12 | 7 | 0 | 0.0045 | 0.0022 |
| 13 | 7 | 0 | 0 | 0 |
| 14 | 7 | 0 | 0 | 0 |
| 15 | 7 | 0 | 0 | 0 |
| 16 | 7 | 0 | 0 | 0 |
| 17 | 6 | 0 | 0 | 0 |
| 18 | 6 | 0 | 0 | 0 |
| 19 | 7 | 0 | 0 | 0 |
| 20 | 8 | 0 | 0 | 0 |
| 21 | 6 | 0 | 0 | 0 |
| 22 | 6 | 0 | 0 | 0 |
| 23 | 6 | 0 | 0 | 0 |
| 24 | 6 | 0 | 0 | 0 |
| 25 | 6 | 0 | 0 | 0 |
| 26 | 6 | 0 | 0 | 0 |
| 27 | 6 | 0 | 0.0001 | 0.0001 |
| 28 | 6 | 0 | 0.0002 | 0.0001 |
| 29 | 7 | 0 | 0.0007 | 0.0003 |
| 30 | 8 | 0 | 0 | 0 |
| 31 | 6 | 0 | 0.0003 | 0.0001 |
| 32 | 6 | 0 | 0.0001 | 0.0001 |
| 33 | 6 | 0.0155 | 0.0001 | 0.0078 |
| 34 | 6 | 0.5 | 0 | 0.25 |
| 35 | 6 | 0.4713 | 0 | 0.2357 |
| 36 | 6 | 0.013 | 0 | 0.0065 |
| 37 | 17 | 0 | 0 | 0 |
| 38 | 17 | 0 | 0 | 0 |
| 39 | 1 | 0 | 0 | 0 |
| 40 | 6 | 0 | 0 | 0 |
| 41 | 0 | 0 | 0 | 0 |
| 42 | 0 | 0 | 0 | 0 |
| 43 | 0 | 0 | 0 | 0 |
| 44 | 0 | 0 | 0 | 0 |
| 45 | 0 | 0 | 0 | 0 |
| 46 | 0 | 0 | 0 | 0 |
| 47 | 0 | 0 | 0 | 0 |
| 48 | 0 | 0 | 0 | 0 |
| 49 | 0 | 0 | 0 | 0 |
| 50 | 0 | 0 | 0 | 0 |
| 51 | 0 | 0.0001 | 0 | 0.0001 |
| 52 | 0 | 0 | 0 | 0 |
| 53 | 0 | 0.0005 | 0.0003 | 0.0005 |
| 54 | 0 | 0 | 0 | 0 |
| 55 | 0 | 0 | 0 | 0 |
| 56 | 0 | 0 | 0 | 0 |

Table S4. FUKUI indices for compound 6d

| **N** | **Z** | **f-** | **f+** | **f0** |
| --- | --- | --- | --- | --- |
| 1 | 8 | 0 | 0 | 0 |
| 2 | 6 | 0 | 0 | 0 |
| 3 | 7 | 0 | 0 | 0 |
| 4 | 6 | 0 | 0 | 0 |
| 5 | 6 | 0 | 0 | 0 |
| 6 | 6 | 0 | 0 | 0 |
| 7 | 6 | 0.0001 | 0 | 0 |
| 8 | 7 | 0.016 | 0 | 0.008 |
| 9 | 8 | 0.0001 | 0 | 0 |
| 10 | 8 | 0.9837 | 0 | 0.4918 |
| 11 | 6 | 0 | 0 | 0 |
| 12 | 6 | 0 | 0 | 0 |
| 13 | 7 | 0 | 0 | 0 |
| 14 | 8 | 0 | 0 | 0 |
| 15 | 8 | 0 | 0 | 0 |
| 16 | 6 | 0 | 0 | 0 |
| 17 | 7 | 0 | 0 | 0 |
| 18 | 7 | 0 | 0 | 0 |
| 19 | 7 | 0 | 0 | 0 |
| 20 | 7 | 0 | 0 | 0 |
| 21 | 6 | 0 | 0 | 0 |
| 22 | 6 | 0 | 0 | 0 |
| 23 | 6 | 0 | 0 | 0 |
| 24 | 6 | 0 | 0 | 0 |
| 25 | 6 | 0 | 0 | 0 |
| 26 | 6 | 0 | 0 | 0 |
| 27 | 7 | 0 | 0 | 0 |
| 28 | 7 | 0 | 0 | 0 |
| 29 | 7 | 0 | 0 | 0 |
| 30 | 7 | 0 | 0 | 0 |
| 31 | 6 | 0 | 0 | 0 |
| 32 | 6 | 0 | 0 | 0 |
| 33 | 7 | 0 | 0 | 0 |
| 34 | 6 | 0 | 0 | 0 |
| 35 | 6 | 0 | 0.0001 | 0 |
| 36 | 7 | 0 | 0 | 0 |
| 37 | 8 | 0 | 0 | 0 |
| 38 | 8 | 0 | 0 | 0 |
| 39 | 6 | 0 | 0.0002 | 0.0001 |
| 40 | 6 | 0 | 0.0043 | 0.0021 |
| 41 | 0 | 0.5581 | 0.2791 | 0.5581 |
| 42 | 0 | 0.0034 | 0.0017 | 0.0034 |
| 43 | 0 | 0.4335 | 0.2167 | 0.4335 |
| 44 | 0 | 0.0003 | 0.0001 | 0.0003 |
| 45 | 0 | 0.0001 | 0 | 0.0001 |
| 46 | 0 | 0 | 0 | 0 |
| 47 | 0 | 0 | 0 | 0 |
| 48 | 0 | 0 | 0 | 0 |
| 49 | 0 | 0 | 0 | 0 |
| 50 | 0 | 0 | 0 | 0 |
| 51 | 0 | 0 | 0 | 0 |
| 52 | 0 | 0 | 0 | 0 |
| 53 | 0 | 0 | 0 | 0 |
| 54 | 0 | 0 | 0 | 0 |
| 55 | 0 | 0 | 0 | 0 |
| 56 | 0 | 0 | 0 | 0 |
| 57 | 0 | 0 | 0 | 0 |
| 58 | 0 | 0 | 0 | 0 |
| 59 | 0 | 0 | 0 | 0 |
| 60 | 0 | 0 | 0 | 0 |
| 61 | 0 | 0 | 0 | 0 |
| 62 | 0 | 0 | 0 | 0 |
| 63 | 0 | 0 | 0 | 0 |
| 64 | 0 | 0 | 0 | 0 |
| 65 | 0 | 0 | 0 | 0 |
| 66 | 0 | 0 | 0 | 0 |
| 67 | 0 | 0 | 0 | 0 |
| 68 | 0 | 0 | 0 | 0 |
| 69 | 0 | 0 | 0 | 0 |
| 70 | 0 | 0 | 0 | 0 |
| 71 | 0 | 0 | 0 | 0 |
| 72 | 0 | 0 | 0 | 0 |
| 73 | 0 | 0 | 0 | 0 |
| 74 | 0 | 0 | 0 | 0 |

Table S5.. FUKUI indices for compound 6e

| **N** | **Z** | **f-** | **f+** | **f0** |
| --- | --- | --- | --- | --- |
| 1 | 6 | 0 | 0 | 1 |
| 2 | 6 | 0 | 0 | 2 |
| 3 | 6 | 0 | 0.0003 | 3 |
| 4 | 6 | 0 | 0.0002 | 4 |
| 5 | 6 | 0 | 0.0002 | 5 |
| 6 | 6 | 0 | 0 | 6 |
| 7 | 6 | 0 | 0.0001 | 7 |
| 8 | 6 | 0 | 0 | 8 |
| 9 | 7 | 0 | 0.004 | 9 |
| 10 | 7 | 0 | 0.4585 | 10 |
| 11 | 7 | 0 | 0.5293 | 11 |
| 12 | 7 | 0 | 0.0046 | 12 |
| 13 | 7 | 0 | 0 | 13 |
| 14 | 7 | 0 | 0 | 14 |
| 15 | 7 | 0 | 0 | 15 |
| 16 | 7 | 0 | 0 | 16 |
| 17 | 6 | 0 | 0 | 17 |
| 18 | 6 | 0 | 0 | 18 |
| 19 | 7 | 0 | 0 | 19 |
| 20 | 8 | 0 | 0 | 20 |
| 21 | 6 | 0 | 0 | 21 |
| 22 | 6 | 0.0133 | 0 | 22 |
| 23 | 6 | 0.477 | 0 | 23 |
| 24 | 6 | 0.4958 | 0 | 24 |
| 25 | 6 | 0.0139 | 0 | 25 |
| 26 | 6 | 0 | 0 | 26 |
| 27 | 6 | 0 | 0.0001 | 27 |
| 28 | 6 | 0 | 0.0002 | 28 |
| 29 | 7 | 0 | 0.0006 | 29 |
| 30 | 8 | 0 | 0 | 30 |
| 31 | 6 | 0 | 0.0003 | 31 |
| 32 | 6 | 0 | 0.0001 | 32 |
| 33 | 6 | 0 | 0.0001 | 33 |
| 34 | 6 | 0 | 0 | 34 |
| 35 | 6 | 0 | 0 | 35 |
| 36 | 6 | 0 | 0 | 36 |
| 37 | 17 | 0 | 0 | 37 |
| 38 | 17 | 0 | 0 | 38 |
| 39 | 1 | 0 | 0 | 39 |
| 40 | 6 | 0 | 0 | 40 |
| 41 | 0 | 0 | 0 | 41 |
| 42 | 0 | 0 | 0 | 42 |
| 43 | 0 | 0 | 0 | 43 |
| 44 | 0 | 0 | 0 | 44 |
| 45 | 0 | 0 | 0 | 45 |
| 46 | 0 | 0 | 0 | 46 |
| 47 | 0 | 0 | 0 | 47 |
| 48 | 0 | 0 | 0 | 48 |
| 49 | 0 | 0 | 0 | 49 |
| 50 | 0 | 0.0001 | 0.0001 | 50 |
| 51 | 0 | 0 | 0 | 51 |
| 52 | 0 | 0.0012 | 0.0006 | 52 |
| 53 | 0 | 0 | 0 | 53 |
| 54 | 0 | 0 | 0 | 54 |
| 55 | 0 | 0 | 0 | 55 |
| 56 | 0 | 0 | 0 | 56 |

Table S6. FUKUI indices for compound 6f

| **N** | **Z** | **f-** | **f+** | **f0** |
| --- | --- | --- | --- | --- |
| 1 | 6 | 0 | 0.0001 | 0 |
| 2 | 6 | 0 | 0.0001 | 0 |
| 3 | 6 | 0 | 0.0005 | 0.0003 |
| 4 | 6 | 0 | 0.0004 | 0.0002 |
| 5 | 6 | 0 | 0.0003 | 0.0001 |
| 6 | 6 | 0 | 0 | 0 |
| 7 | 6 | 0 | 0.0003 | 0.0001 |
| 8 | 6 | 0 | 0 | 0 |
| 9 | 7 | 0.0001 | 0.0039 | 0.002 |
| 10 | 7 | 0 | 0.4526 | 0.2263 |
| 11 | 7 | 0 | 0.5326 | 0.2663 |
| 12 | 7 | 0 | 0.0046 | 0.0023 |
| 13 | 7 | 0 | 0 | 0 |
| 14 | 7 | 0 | 0 | 0 |
| 15 | 7 | 0 | 0 | 0 |
| 16 | 7 | 0 | 0 | 0 |
| 17 | 6 | 0 | 0 | 0 |
| 18 | 6 | 0 | 0 | 0 |
| 19 | 7 | 0 | 0 | 0 |
| 20 | 8 | 0 | 0 | 0 |
| 21 | 6 | 0 | 0 | 0 |
| 22 | 6 | 0 | 0 | 0 |
| 23 | 6 | 0 | 0 | 0 |
| 24 | 6 | 0 | 0 | 0 |
| 25 | 6 | 0 | 0 | 0 |
| 26 | 6 | 0 | 0 | 0 |
| 27 | 6 | 0.0001 | 0.0008 | 0.0005 |
| 28 | 6 | 0.0001 | 0.0005 | 0.0003 |
| 29 | 7 | 0.009 | 0.0009 | 0.005 |
| 30 | 8 | 0 | 0 | 0 |
| 31 | 6 | 0.4997 | 0.0007 | 0.2502 |
| 32 | 6 | 0.0112 | 0.0003 | 0.0057 |
| 33 | 6 | 0 | 0.0002 | 0.0001 |
| 34 | 6 | 0 | 0 | 0 |
| 35 | 6 | 0.0138 | 0 | 0.0069 |
| 36 | 6 | 0.4659 | 0 | 0.233 |
| 37 | 17 | 0 | 0 | 0 |
| 38 | 17 | 0 | 0 | 0 |
| 39 | 1 | 0 | 0 | 0 |
| 40 | 6 | 0 | 0 | 0 |
| 41 | 0 | 0 | 0 | 0 |
| 42 | 0 | 0 | 0 | 0 |
| 43 | 0 | 0 | 0 | 0 |
| 44 | 0 | 0 | 0 | 0 |
| 45 | 0 | 0 | 0 | 0 |
| 46 | 0 | 0 | 0 | 0 |
| 47 | 0 | 0 | 0 | 0 |
| 48 | 0 | 0 | 0 | 0 |
| 49 | 0 | 0 | 0 | 0 |
| 50 | 0 | 0 | 0 | 0 |
| 51 | 0 | 0 | 0 | 0 |
| 52 | 0 | 0.0012 | 0.0006 | 0.0012 |
| 53 | 0 | 0 | 0 | 0 |
| 54 | 0 | 0 | 0 | 0 |
| 55 | 0 | 0 | 0 | 0 |
| 56 | 0 | 0 | 0 | 0 |

**Table S7.** Molecular interactions of synthesized derivatives against targeted protein

| **PDB ID** | **Protein** | **Compound** | **Docking score kcal/mol** | **Hydrogen boning residue** | **Hydrophobic interactions** |
| --- | --- | --- | --- | --- | --- |
| 3DCY | TP53 | 6a | -10.4 | GLN23, ARG203, THR230 | LYS18, ARG10, GLU89, PRO115, LEU100, LEU103, CYS114 |
|  |  | 6b | -11.7 | VAL229, GLN23, ARG203 | PRO115, LEU103, TYR92, CYS 114 |
|  |  | 6c | -9.5 | VAL229, TYR92, GLN 23 | ARG203, ASN17, LEU100 |
|  |  | 6d | -11.8 | ARG61, HIS 198, GLY199,ARG10, ARG104 | ILE22, ARG203, LYS20 |
|  |  | 6e | -11.6 | GLY199, ARG10, HIS11 | PRO115,  CYS114 |
|  |  | 6f | -11.7 | THR230, ARG203, GLN23 | LEU103, CYS114, ILE22, PRO115, TYR92, LYS20, GLU89, ARG10 |
| 1NFI | NF-KAPPA-B P65 | 6a | -9.0 | ARG95 | MET91, GLU92, ARG73, PRO172, LEU173, ARG 174 |
|  |  | 6b | -10.2 | GLN142 | ARG174, ARG73, ARG95, MET91, GLN162 |
|  |  | 6c | -9.2 | THR164, ARG95, ASN137 | GLU92, PRO172, ARG73 |
|  |  | 6d | -10.9 | GLN142, THR164,ASN137, THR136, ARG95, ARG73, VAL163, ARG174, LEU175 | GLU92 |
|  |  | 6e | -10.1 | ARG95 | LEU173, PRO140,PRO177,ARG73 |
|  |  | 6f | -10.7 | GLN142 | ARG73, ARG95, PRO177, ILE145 |
| 3DEI | Caspase-3 | 6a | -9.7 | GLU248 | TRP214, ASN208, TRP206, PHE256, TYR204 |
|  |  | 6b | -9.6 | GLY122 | HIS121, SER205 |
|  |  | 6c | -9.8 | ARG207 | TYR204, LEU168, TRP206, PHE250, ASN208 |
|  |  | 6d | -9.9 | THR62 | PHE250,PHE256, TYR204, |
|  |  | 6e | -9.9 | ARG207 | SER209, TRP206, PHE256, TYR204 |
|  |  | 6f | -10.0 | ARG207 | HIS121, THR62, ARG64, SER63, SER65, SER209, THR166, SER251, PHE250, PHE256, TYR204, LEU168, TRP206, |


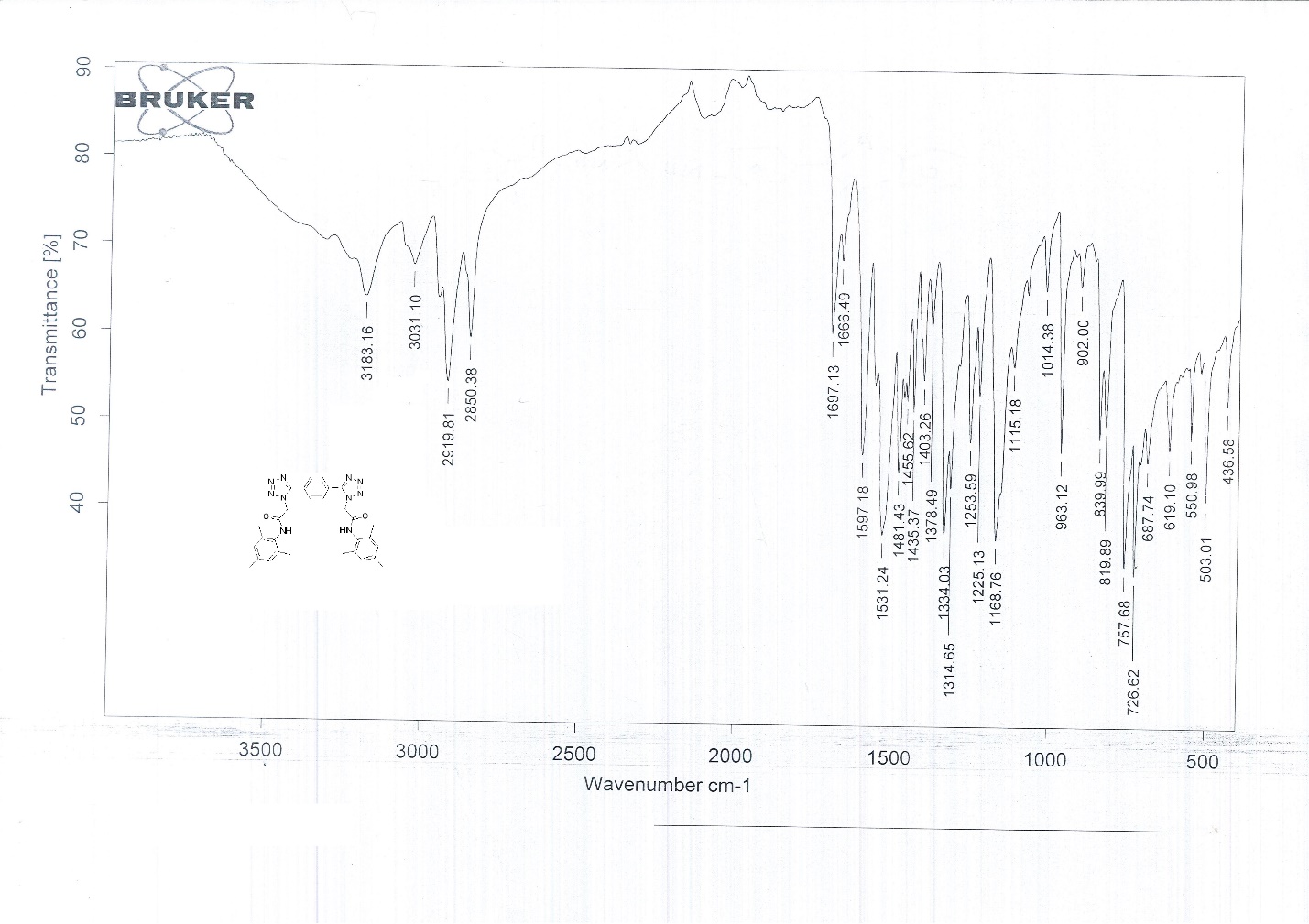


**Figure S1. FTIR Spectra of Compound 6b**


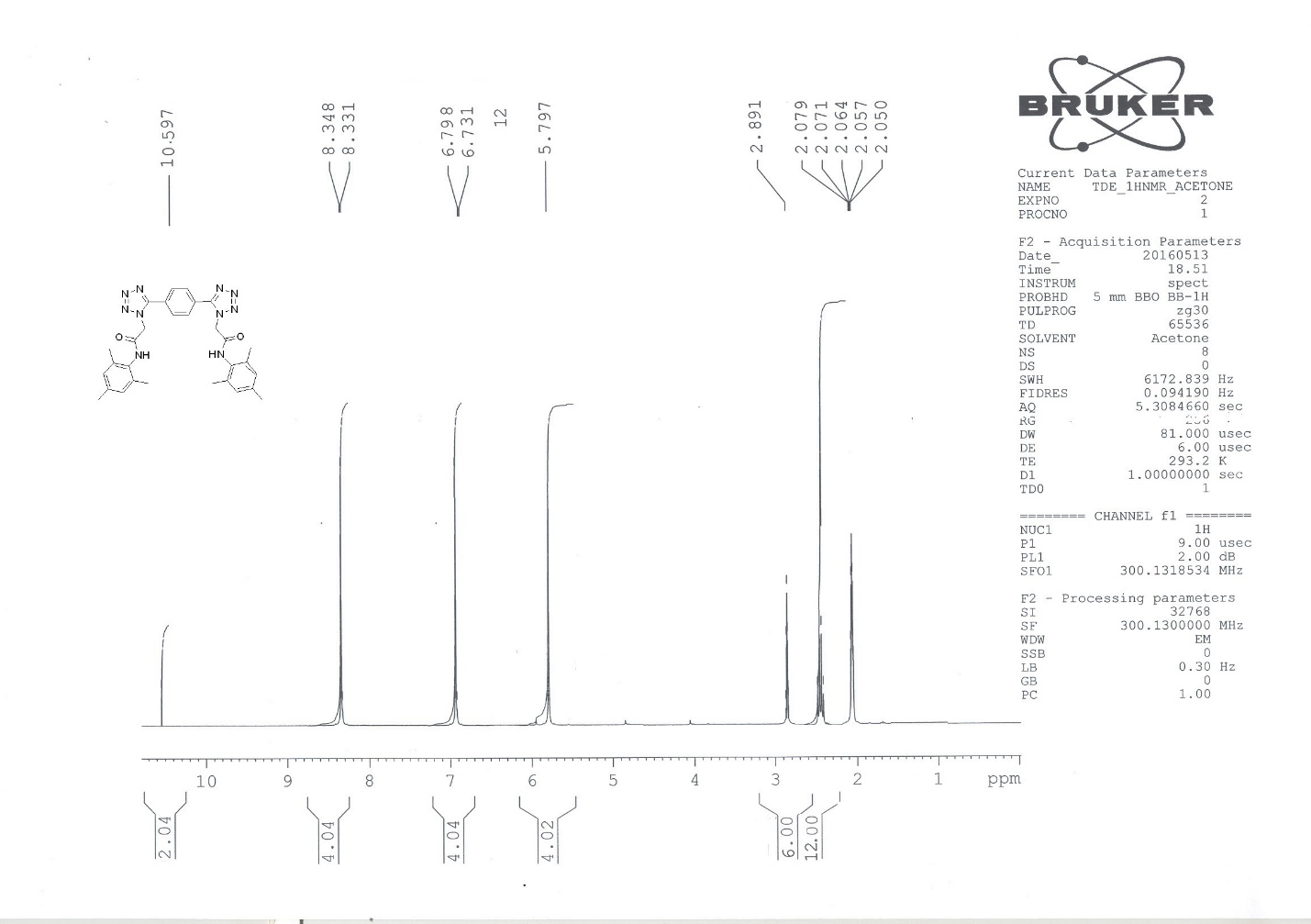


**Figure S2. ^1^H NMR Spectra of Compound 6b**


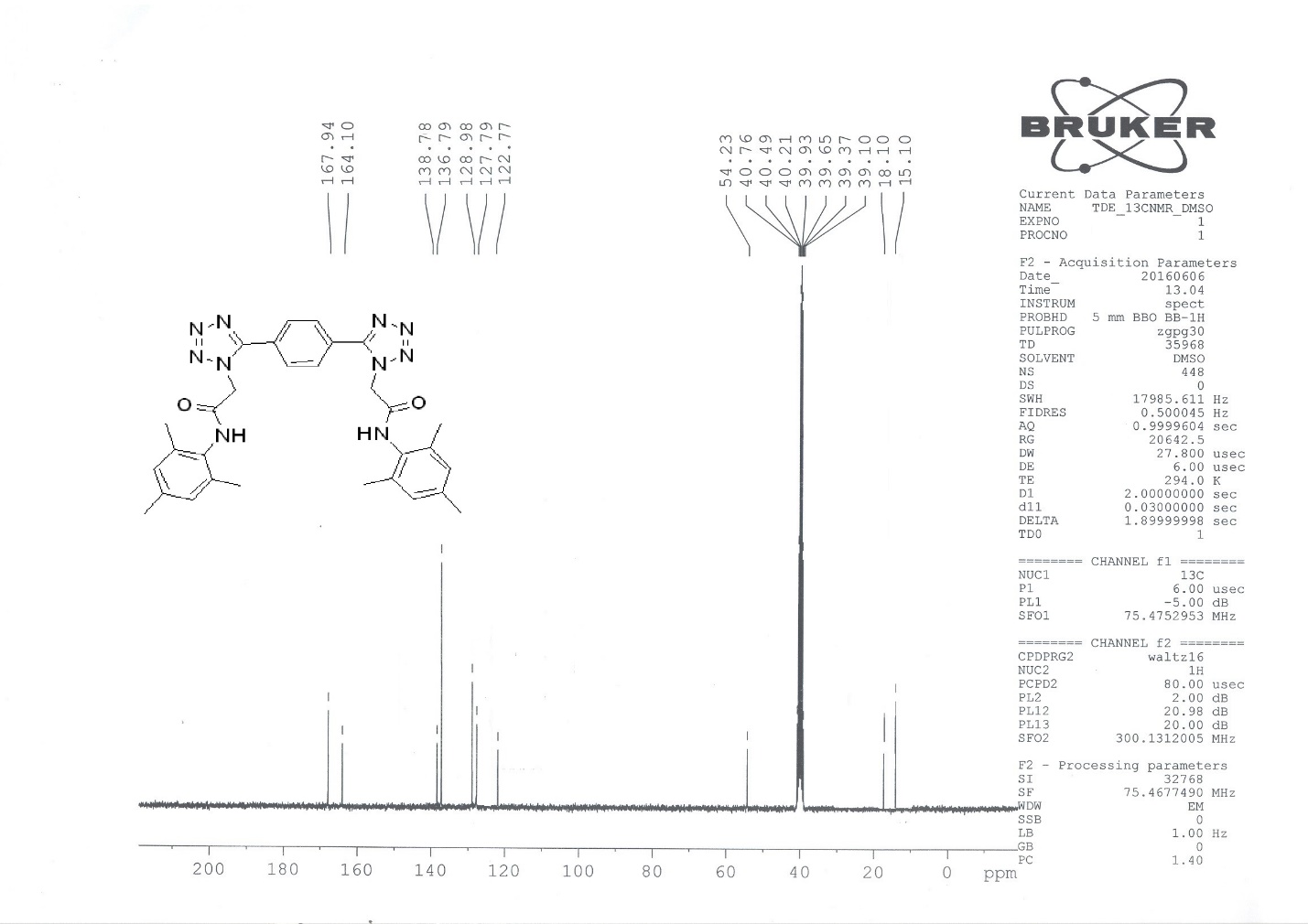


**Figure S3. ^13^C NMR Spectra of Compound 6b**


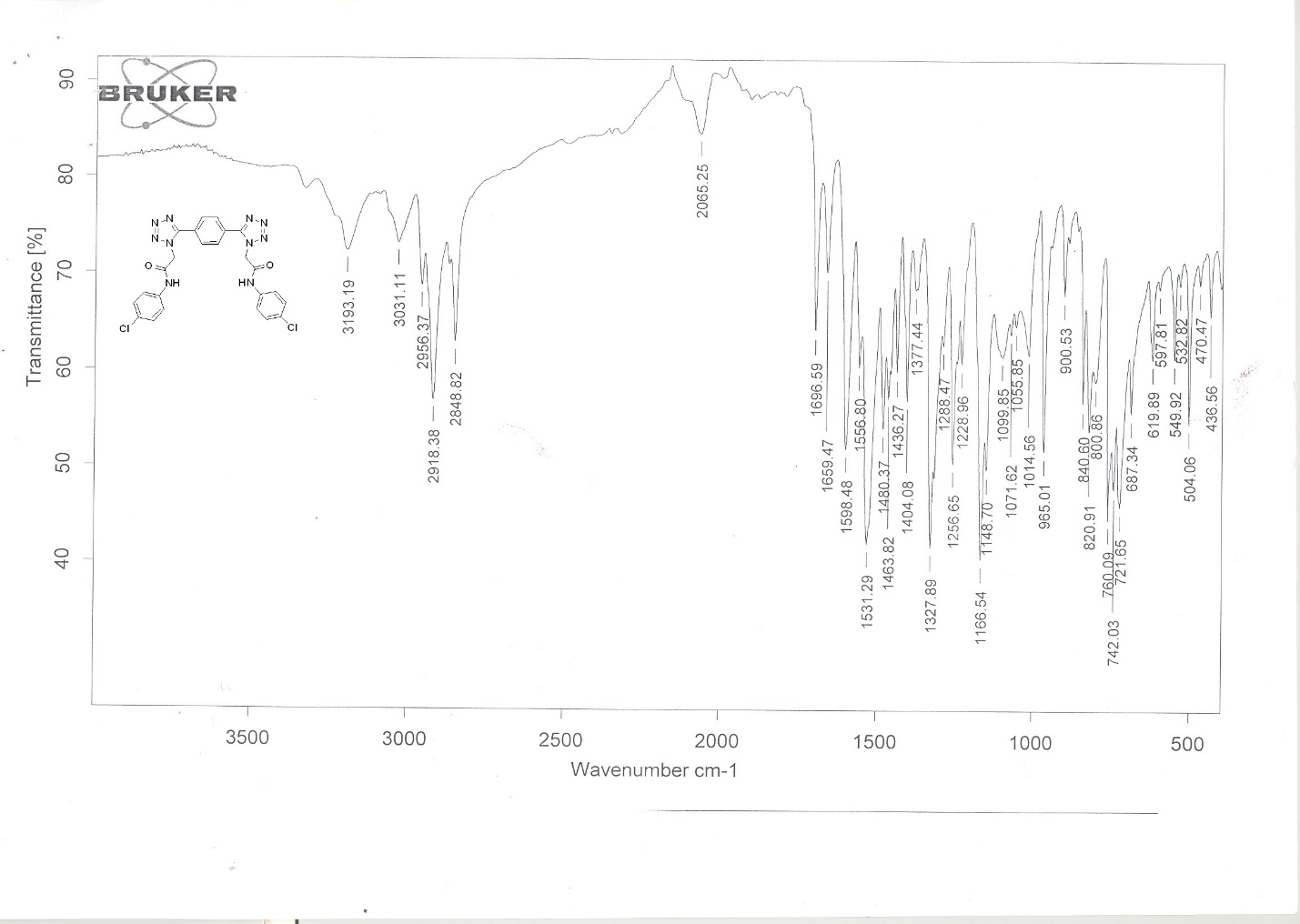


**Figure S4. FTIR Spectra of Compound 6f**


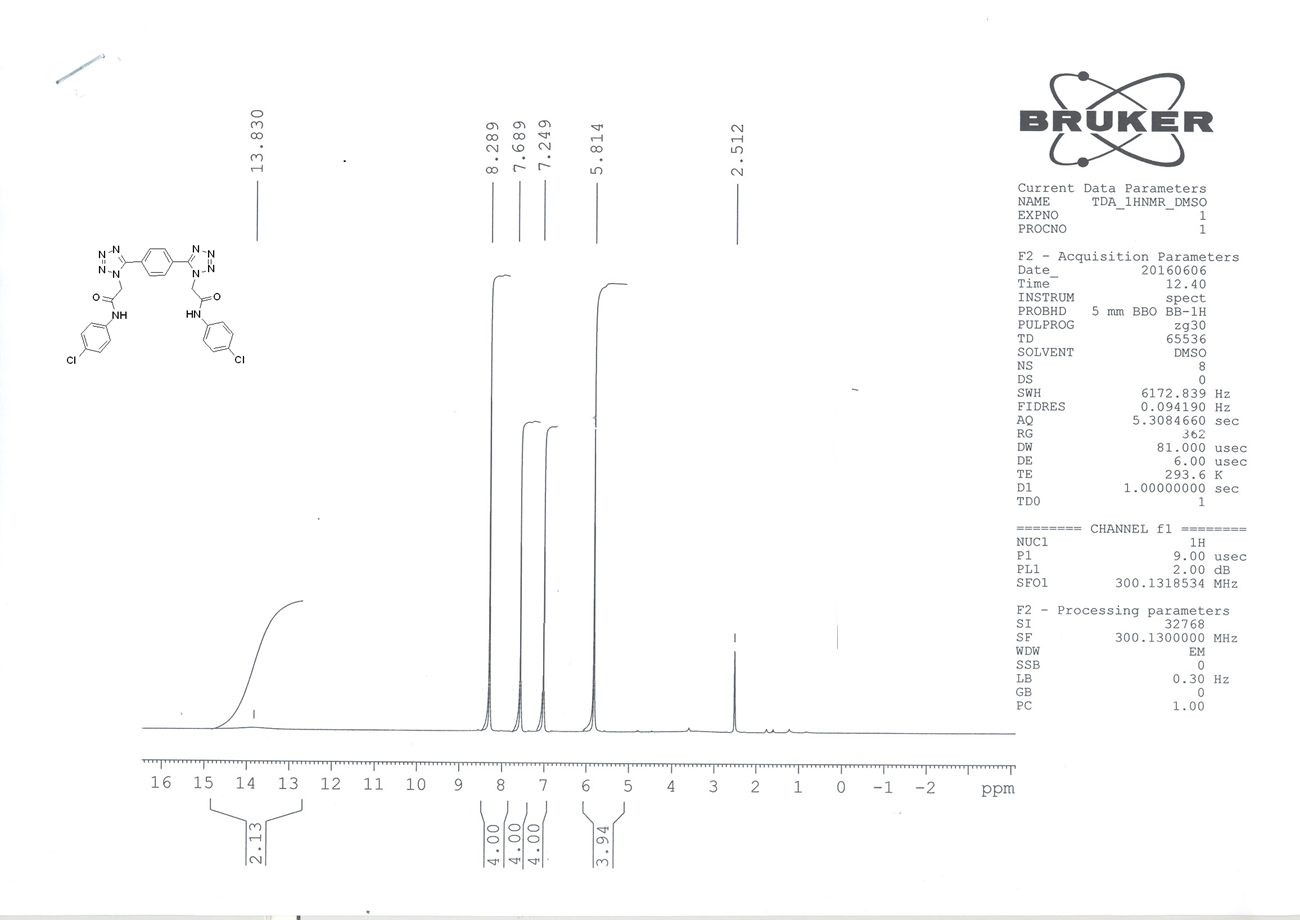


**Figure S5. ^1^H NMR Spectra of Compound 6f**


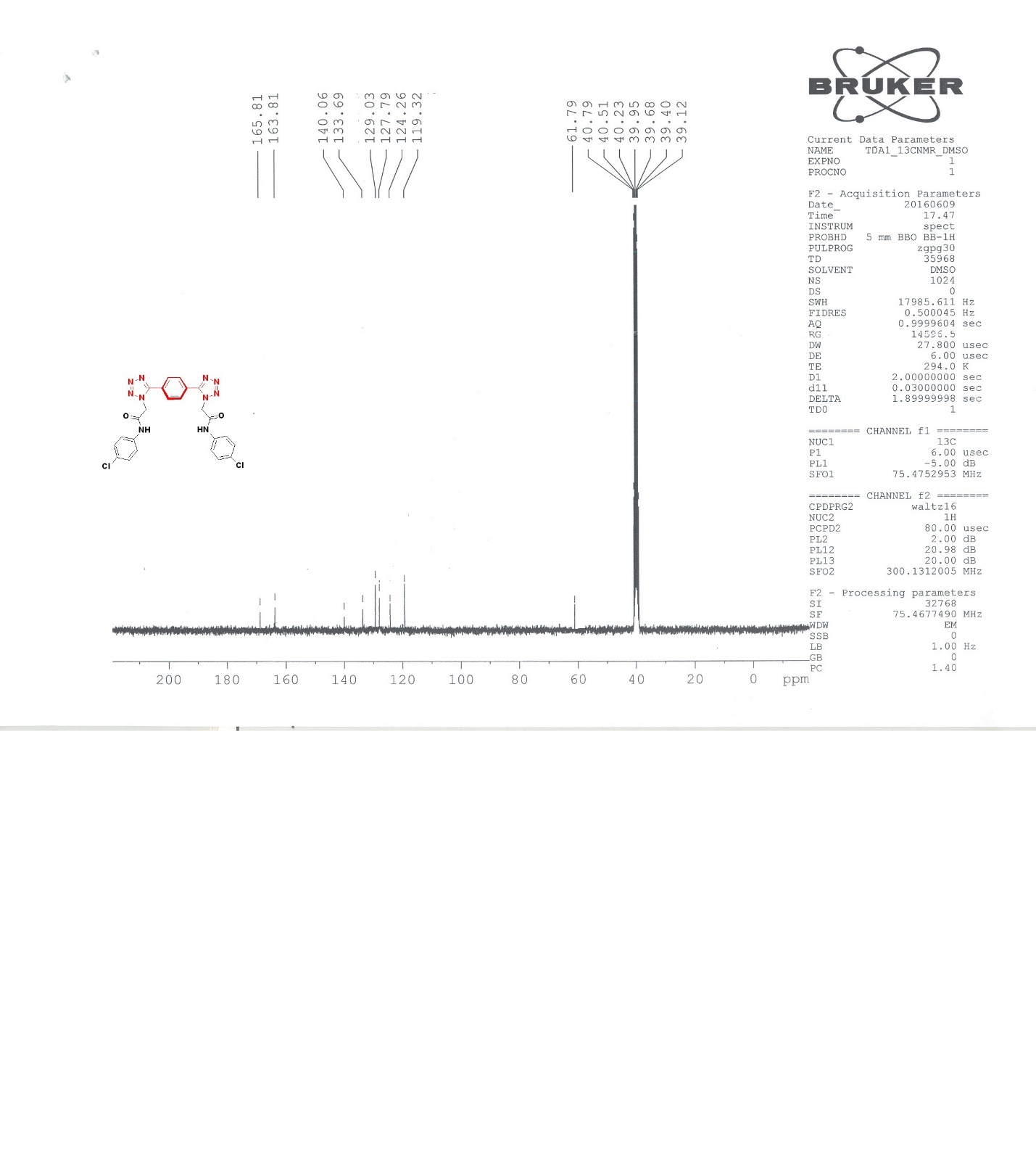


**Figure S6. ^13^C NMR Spectra of Compound 6f**
